# TCMNet: an AI-driven strategy for optimizing traditional Chinese medicine

**DOI:** 10.1186/s13020-026-01360-w

**Published:** 2026-03-31

**Authors:** Shuoyan Tan, Xin Shao, Xuting Zhang, Yizheng Dai, Boli Zhang, Yiyu Cheng, Xiaohui Fan

**Affiliations:** 1 State Key Laboratory of Chinese Medicine Modernization, Pharmaceutical Informatics Institute, College of Pharmaceutical Sciences, Zhejiang University, Hangzhou, 310058 China; 2 State Key Laboratory of Chinese Medicine Modernization, Innovation Center of Yangtze River Delta, Zhejiang University, Jiaxing, 314100 China; 3 State Key Laboratory of Chinese Medicine Modernization, Tianjin University of Traditional Chinese Medicine, Tianjin, 301617 China

**Keywords:** Artificial intelligence (AI), Large language model (LLM), Traditional Chinese medicine (TCM), Herbal formula optimization, Protein–protein interaction (PPI), Parkinson’s disease, Node-weighted networks

## Abstract

**Background:**

Artificial intelligence (AI), particularly large language models (LLMs), have provided powerful tools for systematically modeling the complexity of traditional Chinese medicine (TCM). To overcome the limitations of subjective formula design and unweighted target prioritization, we developed TCMNet, an AI-powered strategy that integrates LLM-assisted disease knowledge mining, protein–protein interaction (PPI) networks, and deep learning-based binding prediction to support herbal formula evaluation and active compounds identification.

**Methods:**

TCMNet integrates AI-guided literature analysis with weighted PPI network evaluation. Parkinson’s disease (PD) was chosen as the representative case study. Disease-associated protein targets were semantically weighted using TCMChat, a TCM-specific LLM that extracts relevant targets from Chinese and English literature. Furthermore, herb-specific information data, such as composition ratios, compound abundance, and compound-protein interaction probabilities, was used to generate weighted herb-related proteins. These weights were incorporated into a PPI network to assign biological weight to each node. Four classical TCM formulas (Tianma Gouteng Decoction, Liuwei Dihuang, Qianzheng San, Dabuyin Wan), the clinically optimized Pingchan Granule (PCG), and their integrative combinations with Western medicine (Levodopa) were systematically evaluated alongside the single-herb *Ginkgo biloba*. Therapeutic relevance was assessed using network-based metrics such as target coverage, Jaccard similarity, and weighted proximity, with statistical significance measured by Z-scores. To validate key active compounds, we employed Boltz-2, a state-of-the-art deep learning method, to predict the binding probabilities between herbal compounds and prioritized PD-associated proteins.

**Results:**

Weighted proximity metrics markedly outperformed unweighted measures across all four evaluated TCM formulas, demonstrating the substantial benefit of integrating node weights. Among the evaluated formulas, Tianma Gouteng Decoction demonstrated superior performance in target coverage and network proximity, aligning well with existing literature. Computational validation on Pingchan Granule (PCG) confirmed that TCMNet successfully captures the therapeutic retention of formula optimization. Furthermore, integrative strategies combining TCM with Levodopa exhibited significantly enhanced network proximity compared to monotherapy, supporting the rationale for combined treatment. Moreover, the case study identified flavonoids and isoflavonoids from *Ginkgo biloba* as the primary bioactive constituents contributing to anti-PD activity. Boltz-2 deep learning predictions further confirmed that flavonoid compounds exhibited significantly higher binding probabilities and affinities toward key PD-associated proteins compared to non-flavonoids, thus validating the results of TCMNet.

**Conclusions:**

By explicitly incorporating protein weights through combining LLM-guided target identification with node-weighted evaluation, TCMNet offers a new AI-driven strategy for optimizing TCM. This approach enables herbal formula evaluation and optimization as well as the identification of bioactive constituents, advancing the modernization of herbal medicine research.

**Supplementary Information:**

The online version contains supplementary material available at 10.1186/s13020-026-01360-w.

## Background

Traditional Chinese medicine (TCM) formulas, which are comprised of multiple herbal constituents, have been widely used in clinical practice for thousands of years. Their multi-components, multi-targets, and comprehensive mechanisms of action offer unique advantages in the prevention and treatment of complex diseases, and have attracted growing global attention in recent years [1]. Due to their inherent complexity, characterized by multiple constituents acting on multiple targets, network-based analytical approaches have become widely utilized in TCM research, providing a valuable entry point for investigating the pharmacological mechanisms of complex herbal formulas [2–4]. However, the design, evaluation, and optimization of TCM formulas have traditionally depended on the empirical knowledge and clinical experience of practitioners. Principles such as syndrome differentiation and formula modification (bian-zheng-jia-jian) remain highly subjective, hindering the standardization, scalability, and modernization of TCM research and new drug development. Therefore, elucidating the molecular basis of TCM and developing objective, quantitative evaluation metrics have become central scientific challenges in the field. There is an urgent need for data-driven methodologies that can systematically guide formula refinement and simplification.

The rapid development of artificial intelligence (AI) [5], high-throughput omics [6, 7], and network pharmacology [8–10] have laid the foundation for modernizing TCM research [11, 12]. Specialized tools such as BATMAN-TCM2.0 [13] and TCM-specific large language models (LLMs) [14], have provided crucial foundations for constructing comprehensive herb-compound-target-disease networks and facilitating intelligent mining of classical texts and clinical records. Moreover, the network science paradigm [15–17] provides a powerful theoretical framework, positing that drugs treat diseases by modulating specific disease modules within the human interactome network. These advances allow for a node-weighted analysis of how multi-components formulas intervene in complex biological networks [18].

Despite these advances, several critical limitations remain in current network-based studies [19]. Among these, three major issues remain particularly unaddressed and form the focus of the present study: (i) Most studies fail to differentiate the relative priority of targets, lacking weighted algorithms that integrate multi-source data such as semantic evidence from literature. (ii) Conventional methods treat all ingredient-target interactions as binary edges, failing to account for critical pharmacological factors such as herb dosage, compound abundance, and binding probability. (iii) There is a scarcity of systematic methodologies to objectively identify core bioactive constituents from complex formulas. Addressing these gaps is critical for both scientific advancement and for increasing the international recognition of TCM. Overcoming these limitations requires integrative computational strategies capable of systematically evaluating the contributions of individual herbs and their constituent compounds within complex prescriptions, thereby substantially advancing the modernization, standardization, and rationalization of TCM practice and research.

To overcome these challenges, we propose TCMNet, an AI-driven strategy specifically for node-weighted network analysis. The core innovation of TCMNet is the establishment of a multi-source, quantitative node-weighted scoring system. TCMNet systematically evaluates the relative priority of disease-associated targets and herbal constituents by integrating data-driven weighting approaches, including LLM-assisted literature mining and biochemical feature quantification. This framework enables quantitative formula optimization, simplification, and identification of bioactive core constituents. This approach explicitly integrates disease-associated target weight from multiple databases and systematically quantifies herb-related protein relevance using detailed biochemical data, including composition ratios of herb, compound abundance, and predicted compound-protein interaction probabilities. In this study, we apply TCMNet to Parkinson’s disease (PD) as a representative complex disorder, specifically evaluating four widely utilized TCM formulas: Tianma Gouteng Decoction (TGD), Liuwei Dihuang (LWDH), Qianzheng San (QZS), and Dabuyin Wan (DBYW). Additionally, we extend the analysis to a single-herb case study of *Ginkgo biloba* to show that, beyond multi-herb formulas, TCMNet can identify and prioritize key bioactive constituents. Our findings demonstrate that integrating node weight into network models significantly enhances predictive accuracy and interpretability. Thus, TCMNet provides a robust, generalizable computational framework for the objective evaluation, optimization, and mechanistic elucidation of TCM formulas, and may substantially accelerate the scientific modernization and global acceptance of TCM.

## Methods

### LLM-assisted literature mining and target extraction

Formula research popularity was assessed through systematic literature searches in the CNKI and SciFinder databases, using a combination of formula names and “Parkinson’s disease” as keywords. Similarly, disease-associated targets were explored by searching databases with disease names and their corresponding TCM syndrome terms. To extract disease-relevant entities from unstructured literature, full texts were automatically retrieved using customized web-crawling scripts. The processed textual content was then input into TCMChat [14], a TCM-specific LLM, via prompt engineering strategies. The model output was parsed to extract named entities corresponding to protein targets, which were subsequently filtered and classified using regular expression-based rules. Specifically, over 30,000 articles were processed using a distributed pipeline with text chunking, and mapped all extracted targets to official HGNC Gene Symbols to resolve naming redundancies. We utilized the updated TCMChat1.5 model, which has been fine-tuned for enhanced entity recognition capabilities. Specifically, TCMChat1.5 improves upon version 1.0 [14] by adopting Qwen as the foundation model and incorporating additional training data focused on literature entities. To rigorously validate extraction accuracy and assess potential false-positive rates, we conducted a comparative benchmark using the TCM-NER dataset, a human-curated ground truth set containing 2,480 manually annotated samples retrieved from the AliTianchi platform (https://tianchi.aliyun.com/dataset/86819). We evaluated a total of five models, including four external baselines (BERT-CRF, GPT-3.5-turbo [20], HuatuoGPT [21], and Qwen1.5-7B) and two versions of our domain-specific model (TCMChat1.0 and TCMChat1.5). Entity extraction performance was quantified using Precision, Recall, and F1-score.

To quantify the semantic relevance of each target, we calculated an LLM-derived score (LLMscore). First, the frequency (Count_i_) of each unique target appearing across the entire corpus was tallied. To align with the scales of other database scores, this raw frequency was normalized by dividing it by the maximum frequency observed in the dataset, thereby scaling the score to a range of [0, 1]:

1 $${LLMscore}_{i}=\frac{{Count}_{i}}{\mathrm{max}(Count)}$$

This normalized score quantitatively reflects the frequency and contextual relevance of each target based on automated literature mining.

### Disease-associated proteins collection and weight integration

This study aimed to systematically identify and prioritize protein targets associated with complex diseases, using Parkinson’s disease (PD) as the representative case. We retrieved disease-associated targets by searching each database using “Parkinson’s disease” as the specific keyword. Protein targets were obtained from four major public databases: GeneCards [22], DisGeNET [23], Comparative Toxicogenomics Database (CTD) [24] and the Therapeutic Target Database (TTD) [25]. All data from these sources were accessed on April 21, 2025. For data consistency, we restricted all datasets to protein-coding genes. Target identifiers were standardized to official HGNC Gene Symbols to resolve naming variations. Each database provided different scoring metrics reflecting target-disease associations. To ensure comparability across disparate scales and metrics provided by these databases, we standardized all scores to the interval (0, 1]. For CTD and GeneCards, we applied Min–Max scaling to normalize the original scores. Since this method maps the lowest observed scores to zero, we replaced these zero values with a small epsilon (ϵ = 10^–5^) to prevent them from nullifying the final weight during multiplicative integration. The ScoreGDA metrics from the DisGeNET database, which are standardized between 0 and 1 by definition, were utilized directly. For TTD, a qualitative database, we assigned a score of 1.0 to listed targets and a minimal value (ϵ_missing_ = 10^–20^) to unlisted ones. Similarly, this minimal value [10–20] was imputed for missing targets in the other databases. To further enrich target prioritization with semantic and literature-derived relevance, we integrated the LLMscore (calculated as described in the previous section). The integrated weight (TargetScore) for each target was calculated as the product of the normalized scores from all four databases and the LLMscore. We adopted this multiplicative approach to prioritize consensus targets supported by multiple independent lines of evidence:

2 $${targetscore}_{i}={CTDScore}_{i}\times {DisGeNETscore}_{i}\times {Genecardsscore}_{i}\times {TTDscore}_{i}\times {LLMscore}_{i}$$

After normalization, PD-associated proteins were ranked by their integrated scores, and Top100–Top1000 subsets (in increments of 100) were selected for downstream prioritization and comparative evaluation.

### Comparison with additive integration strategy

To assess the robustness of our multiplicative scoring system, we constructed an alternative additive model where the integrated weight was calculated as the sum of normalized scores from the five sources (CTD, DisGeNET, GeneCards, TTD and LLM). Missing scores were imputed as zero. We compared the target rankings derived from both schemes using Jaccard similarity indices and Spearman’s rank correlation coefficients across varying Top-N thresholds (N = 50–500). We evaluated the stability of target rankings against data noise by introducing random perturbations.

### External validation via single-cell RNA sequencing (scRNA-seq) data

To biologically validate the prioritized disease-associated proteins, we utilized a single-cell transcriptomic dataset of human PD prefrontal cortex [26] (Accession: GSE202210). The raw data were processed using the Seurat package (v5.3.0) in R (v4.4.2). We identified differentially expressed genes (DEGs) between PD patients and healthy controls for both the aggregate cell population and six specific cell types (astrocytes, excitatory neurons, inhibitory neurons, microglia, oligodendrocytes, and oligodendrocyte precursor cells). DEGs were defined based on an adjusted P-value < 0.05 (Wilcoxon rank-sum test) and an absolute log2 fold change ≥ 0.25. The biological relevance of our computational strategy was quantified by calculating the Jaccard similarity index between the predicted high-weight targets (Top N) and the identified DEGs for each cell type.

### Herb-related protein identification and weight calculation

Classic TCM formulas widely used for PD treatment were selected for this study, namely Qianzheng San (QZS), Dabuyin Wan (DBYW), Liuwei Dihuang (LWDH), Tianma Gouteng Decoction (TGD), and Pingchan Granule (PCG) [27]. Details of formula composition and composition ratios are provided in Supplementary Table S2. Herbal ingredients and target data were obtained from the BATMAN-TCM2.0 database [13], complemented by herbal compound abundance data from the Meta-TCM database (an internal, unpublished laboratory database). To comprehensively quantify herb-related protein weights, three progressive calculation methods were employed. Method 1 calculated target weights solely based on herbal proportions. Method 2 extended this approach by incorporating both herb proportions and chemical composition data but did not account for compound-target interaction probabilities. Method 3, the most comprehensive strategy, integrated herb proportions, chemical compositions, and compound-target interaction probabilities. The calculation for the integrated weight of a specific protein target j (W_j_^raw^) in Method 3 is defined as the sum of weighted contributions from all interacting compounds across all herbs:

3 $${W}_{j}^{raw}=\sum_{h\in Herbs}\sum_{c\in compounds}({R}_{h}\times {A}_{c,h}\times P(c,j))$$

R_h_ is the normalized dosage ratio of the h-th herb in the formula (∑Rh = 1). A_c,h_ is the relative abundance of the c-th compound in the h-th herb (derived from Meta-TCM; set to 1.0 for Method 1). Pc,j represents the interaction confidence score derived from BATMAN-TCM 2.0. For experimentally validated interactions, a definitive probability (P = 1.0) was assigned. For predicted interactions, Pc,j corresponds to the similarity-based confidence score (ranging < 1.0) calculated by BATMAN’s algorithm, which evaluates multidimensional similarities (e.g., chemical structure, protein sequence) between the query pair and known interaction seeds. For Methods 1 and 2, all interactions were treated as P = 1.0.

To ensure comparability across metrics and maintain network connectivity, raw weights were normalized to the interval $(\mathrm{0,1}]$ using a modified Max–Min scaling approach with a smoothing factor. To prevent the elimination of potential proteins (where weight = 0), we introduced a small epsilon ($\epsilon ={10}^{-6}$). The final normalized weight W_J_^norm^ was calculated as:

4 $${W}_{target}^{norm}=\frac{{W}_{target}^{raw}-\mathrm{min}({W}^{raw})}{\mathrm{max}({W}^{\left(raw\right)})-\mathrm{min}({W}^{raw})}\times \left(1-\epsilon \right)+\epsilon$$

This procedure ensures that all protein weights are strictly positive and fall within (0,1], facilitating robust comparative analyses across herbs and formulas.

### Protein–Protein Interaction (PPI) Network

We utilized the high-quality human PPI network established by Cheng et al. [28], which comprises 16,677 proteins and 243,603 undirected interactions. This interactome integrates physical interaction data derived from curated databases, including IntAct, InnateDB, PINA, HPRD, BioGRID, HI-II-14_Net, PhosphositePlus, KinomeNetworkX, Instruct, and MINT. The original network data provided proteins identified by NCBI Entrez IDs. To ensure topological consistency with our disease-associated and herb-related protein datasets, protein identifiers were standardized by mapping the original NCBI Entrez IDs to official HGNC Gene Symbols using the NCBI *gene2accession* annotation file, and any resulting duplicate interactions were merged.

### Network evaluation metrics

While topological metrics such as degree and betweenness centrality reflect the static weight of individual nodes, they are insufficient to quantify the systemic relationship between an herbal formula and a disease module. To systematically evaluate the therapeutic potential of TCM formulas, which act as multi-components agents targeting complex disease-associated subnetworks, we selected a set of biologically informed network metrics instead of simple centrality measures. These metrics were chosen to align with the “network medicine” paradigm [15], quantifying both the systemic breadth of target engagement and the topological proximity of therapeutic intervention. To quantify the extent of direct intervention and the indirect regulatory reach of herbal formulas within the biological network, we defined two coverage metrics. The first, coverage_overlap, measures the fraction of disease-associated proteins that are also targeted by the herbal constituents, reflecting direct overlap between the two sets. The second, coverage_directlink, evaluates the proportion of disease-associated proteins that are directly reachable from herb-related proteins through at least one path within the protein–protein interaction (PPI) network, thereby capturing potential indirect regulatory relationships.

5 $${Coverage}_{overlap}=\frac{|H\cap D|}{|D|}$$

where *H* denotes the set of herb-related proteins, and *D* represents the set of disease-associated proteins.

6 $${Coverage}_{directlink}=\frac{|D\cap (H\cup N(H))|}{|D|}$$

where* N(H)* represents the set of first-degree neighbors of herb-related proteins within the PPI network.

Additionally, the Jaccard similarity index was computed to quantify the overlap between herbal and disease-associated protein sets. The Jaccard index evaluates the degree of similarity between the two sets as follows:

7 $$Jaccard\left(H,D\right)=\frac{|H\cap D|}{|H\cup D|}$$

In this study, we used the proximity metric to quantify the capability of herb-related proteins in modulating disease-associated proteins within the protein–protein interaction (PPI) network. The network proximity approach has been previously employed to investigate drug-disease associations and related network relationships [15].

The unweighted proximity between a set of herb-related  proteins *H* and disease-associated proteins *D* was defined as the average shortest path length between all pairs of nodes:

8 $$d\left(H,D\right)=\frac{1}{H}\sum_{h\in H}\underset{d\in D}{\mathrm{min}}\left(dist(h,d)\right)$$

where dist(ℎ,*d*) represents the unweighted shortest path length between nodes ℎ and *d* in the PPI network. Shortest path lengths were computed using the Breadth-First Search (BFS) algorithm implemented in the NetworkX package.

To incorporate the biological weight of specific nodes, we assigned weights to each protein node—designated as *w*ℎ for herb-related protein node ℎ and *wd* for disease-associated protein node *d*. The weighted proximity is defined as:

9 $${d}_{(H,D)}^{weighted}=\frac{\sum_{h\in H}\sum_{d\in D}dist(h,d){w}_{h}{w}_{d}}{\sum_{h\in H}\sum_{d\in D}{w}_{h}{w}_{d}}$$

This weighting strategy prioritized node pairs of higher biological relevance by scaling their distance contributions by the product of their corresponding weights.

To assess the statistical significance of the observed proximity values, both weighted and unweighted proximity metrics were compared against null distributions obtained from 500 randomized sets of nodes of equal size. The random sampling was conducted in a degree-aware manner, preserving the topological characteristics of the PPI network. The statistical significance was quantified using the Z-score:

10 $$Z score=\frac{{S}_{obs}-{\mu }_{rand}}{{\sigma }_{rand}}$$

where *S*_obs_ is the observed proximity score, and μ_rand_, σ_rand_ are the mean and standard deviation of proximity scores computed from randomized node sets.

To evaluate the robustness and stability of the calculated protein node weights against random fluctuations or noise, we implemented a weight perturbation sensitivity analysis. This method assesses how resilient the rank ordering of protein targets is when the original weights are subjected to varying levels of random noise. Let w_i_ denote the original normalized weight assigned to protein node *i*. For a given perturbation coefficient α∊[0, 1], the perturbed weight is defined as a convex combination of the original weight and a random value r_i_ drawn from a uniform distribution:

11 $${w}_{i}^{(\alpha )}=\left(1-\alpha \right){w}_{i}+\alpha {r}_{i}$$

Here, α = 0 corresponds to no perturbation (original weights), while α = 1 represents full randomization of the weights. For each perturbation level α, we performed 100 independent simulations using a fixed random seed to ensure reproducibility. We then calculated the mean Spearman rank correlation coefficient Rs(α) between the original weight ranking and the perturbed ranking across all protein nodes, thereby quantifying the sensitivity of our weighting scheme to perturbation.

### Deep learning-based ligand–protein binding affinity prediction

The binding affinities and probability between small molecules and protein targets were predicted using the Boltz-2 model [29], a state-of-the-art structural biology foundation model developed by MIT. Boltz-2 was selected for its superior performance on rigorous external benchmarks, including the CASP16 affinity challenge and the FEP^+^ benchmark. It has been demonstrated to approach the accuracy of alchemical Free Energy Perturbation (FEP) simulations and significantly outperform traditional docking methods (e.g., Glide, Vina) in ranking active compounds. The model operates on protein sequences and molecular SMILES representations. Compound SMILES were obtained from the PubChem database, while protein sequences were retrieved from UniProt. Due to the excessive length of their amino acid sequences, kinase domains were specifically used for IGF1R (residues 961–1270) and LRRK2 (residues 1879–2140); all other proteins were analyzed using their full-length sequences.

## Results

### Overview of the TCMNet workflow

**Fig. 1:**
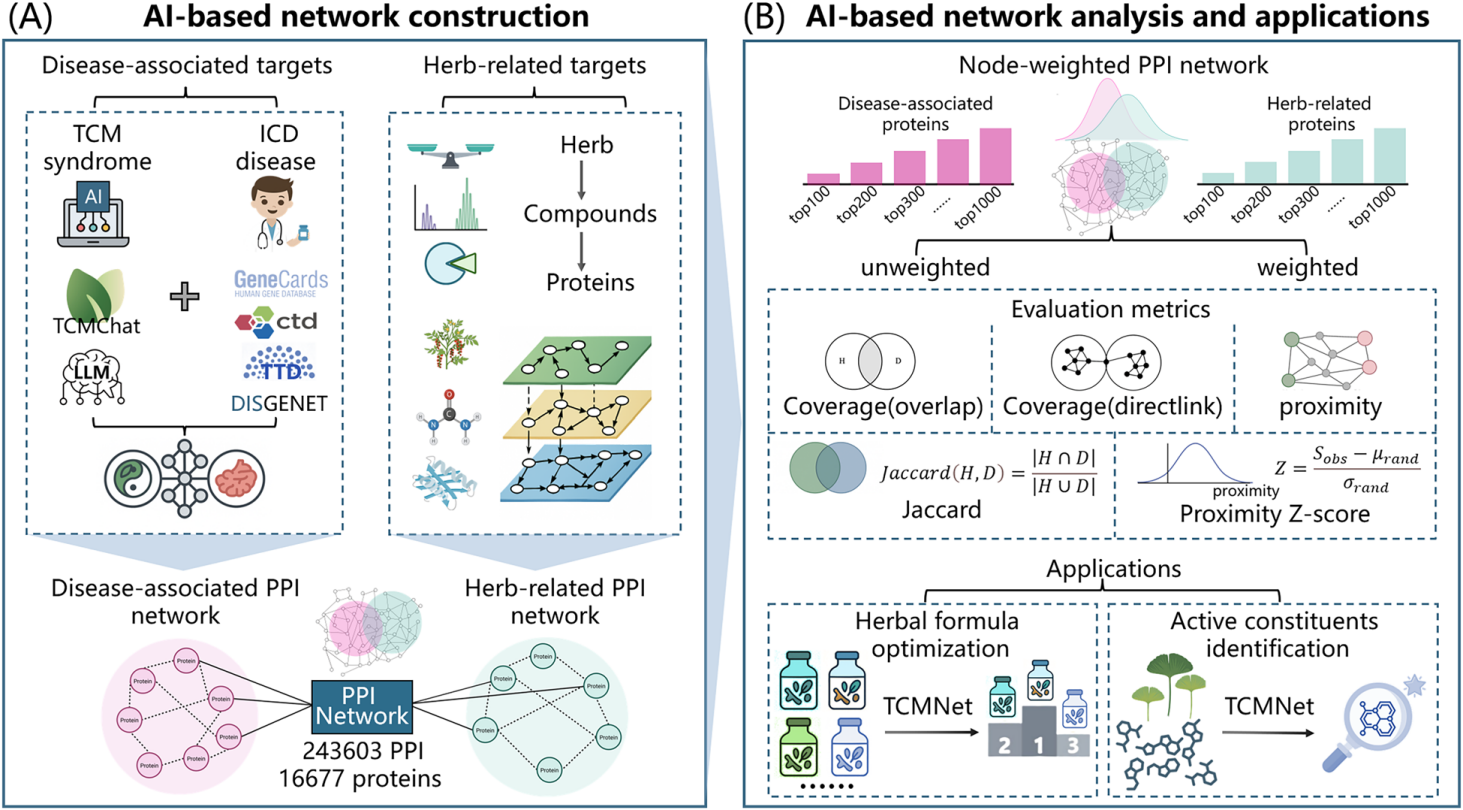
Workflow of the TCMNet strategy. **A** AI-based network construction, including LLM-assisted knowledge mining and protein–protein interaction (PPI) network construction. **B** AI-based network analysis and applications

The TCMNet strategy integrates node-weighted network pharmacology to systematically prioritize and evaluate the therapeutic potential of TCM formulations for complex diseases. As illustrated in Fig. 1, the overall workflow consists of two major steps: AI-based network construction and AI-based network analysis and application.

First, disease-associated protein targets were systematically integrated through LLM-assisted knowledge mining. Specifically, disease-associated proteins were identified not only by automated knowledge extraction using our in-house TCM-specific large language model, TCMChat, but also by aggregating information from authoritative databases including DisGeNET, GeneCards, CTD, and TTD. By combining these multiple sources, the strategy ensures comprehensive inclusion of targets relevant to both Western and traditional Chinese medical concepts and symptoms. Subsequently, information such as herbal composition ratios, compound abundance, and compound-target interaction probabilities was integrated to generate herb-related proteins and their weights. All identified targets were then mapped onto a large-scale protein–protein interaction (PPI) network containing 16,677 proteins and 243,603 interactions, which completed the network construction and weighting. Based on the constructed and weighted PPI network, we calculated a set of network metrics, including coverage_overlap, coverage_directlink, Jaccard similarity, network proximity, and corresponding Z-scores, to systematically evaluate the disease-intervention potential of different TCM formulas. This workflow not only quantifies the impact of herbal components on disease networks, but also supports the prioritization of core bioactive ingredients and the optimization of herbal formulas.

To validate this strategy, we selected Parkinson’s disease (PD) and assessed four classical TCM formulas with reported PD relevance: Qianzheng San (QZS), Dabuyin Wan (DBYW), Liuwei Dihuang (LWDH), and Tianma Gouteng Decoction (TGD).

### Large language model (LLM)-guided discovery and prioritization of disease-associated proteins

Parkinson’s disease (PD) is a progressive neurodegenerative disorder with complex and largely unknown etiology, characterized by the degeneration of dopaminergic neurons in the substantia nigra. Given the multifactorial nature of PD and the lack of curative treatments, multi-target therapeutic strategies, such as those offered by traditional Chinese medicine (TCM), have attracted considerable research interest. Therefore, selecting PD as the disease model for this study is particularly appropriate, as it allows for a comprehensive evaluation of the network-based, multi-target advantages of TCM interventions.

To comprehensively identify and prioritize disease-associated protein targets, we implemented an AI-powered literature mining approach in conjunction with biomedical databases. Specifically, a total of 27,470 Chinese and 4749 English PD-related articles were automatically retrieved using web-crawling scripts and analyzed via TCMChat, a TCM-specific large language model (LLM). This enabled automated extraction and scoring of PD-associated targets from both classical and modern literature, resulting in a literature-derived semantic relevance score (LLMscore) for each target. Before prioritizing targets, we validated the reliability of our mining workflow. Quantitative benchmarking on the TCM-NER dataset revealed that TCMChat1.5 achieved robust performance with an F1-score of 0.910, significantly outperforming other open-source models (e.g., HuatuoGPT, F1 = 0.743) and showing comparable accuracy to the state-of-the-art GPT-3.5-turbo (F1 = 0.914) (Table S1). Crucially, the model demonstrated a high Precision of 0.977, indicating a minimal false-positive rate (~ 2.3%), which effectively limits the inclusion of irrelevant noise entities into the downstream network analysis.

In parallel, PD-associated protein targets were systematically collected from four widely recognized biomedical databases: CTD, GeneCards, DisGeNET, and TTD. As shown in Fig. S1A, these databases provide partially overlapping but complementary scope. To systematically prioritize these targets, we defined an integrated weight, termed TargetScore, which represents the Integrated Consensus Score of a protein’s association with disease. We calculated this score by multiplying normalized scores from the four databases and the LLMscore (Fig. S1B).

We deliberately adopted this multiplicative integration strategy rather than an additive one to act as a “consensus filter”, prioritizing targets supported by multiple independent lines of evidence while penalizing those lacking consistent support. To validate this scoring rationale, we systematically compared the multiplicative strategy against an additive scheme. The two methods demonstrated substantial consistency in prioritizing top-ranked targets, maintaining high Jaccard overlap and Spearman correlations across Top 50–500 subsets (Table S2). Sensitivity analysis further confirmed that both schemes preserved robust global ranking structures under random weight perturbation, although the multiplicative model showed slightly higher sensitivity to noise due to its stricter consensus logic (Fig. S2AB). Crucially, biological benchmarking against external PD scRNA-seq data revealed that the multiplicative approach achieved superior alignment with disease transcriptomic signatures (Fig. S2C). Notably, it yielded higher target overlap in microglial cells, key drivers of PD neuroinflammation [30], thereby demonstrating enhanced capability to capture cell-type-specific pathological signals. These results confirm that the multiplicative strategy effectively prioritizes robust, biologically validated consensus targets.

Based on this robust and validated scoring system, high-weight targets, such as SNCA (α-synuclein), TH (tyrosine hydroxylase), LRRK2 (leucine-rich repeat kinase 2), DDC (dopa decarboxylase) [31], and MAPT (microtubule-associated protein tau), were ranked highest, which is consistent with current knowledge of PD pathophysiology [32, 33] (Table S4). Consensus analysis revealed that the Top 8 targets are supported by all five data sources, and the Top 167 by at least four, confirming high data reliability. These integrated scores enabled ranked selection of Top-N PD-associated proteins (e.g., Top100 to Top1000) for subsequent network-based analyses. To mitigate selection bias, we analyzed the full spectrum from these high-confidence “core disease-associated proteins” (Top 100 +) to the broader “disease module” (Top 1000). This multi-scale approach serves as a sensitivity analysis, ensuring that our findings are robust and not artifacts of specific arbitrary thresholds.

### Herb-related protein weighting and comparative evaluation

**Fig. 2:**
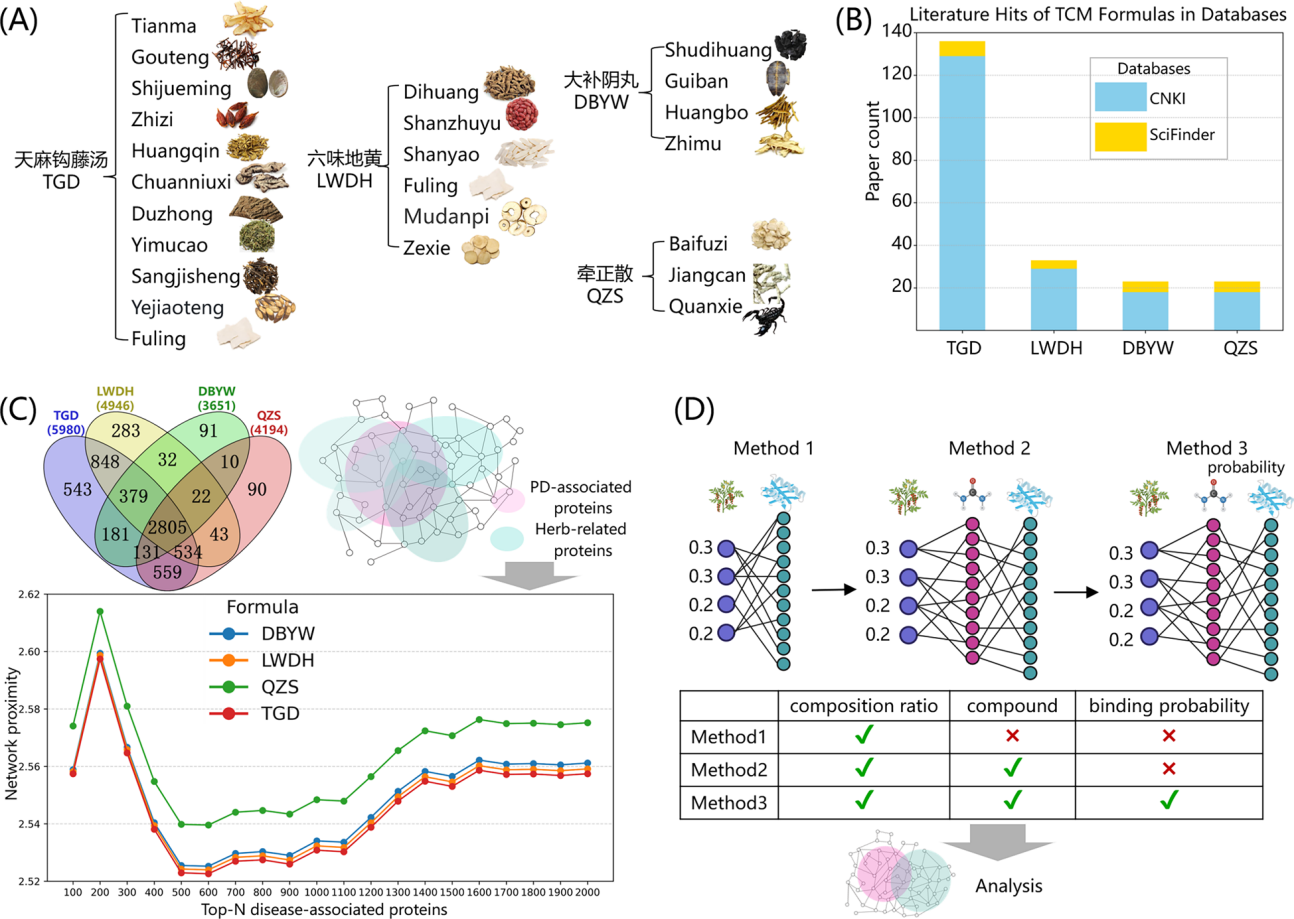
**A** Composition of four classical PD-related TCM formulas (TGD, LWDH, DBYW, QZS). **B** literature popularity of four TCM formulas (TGD, LWDH, DBYW, QZS). **C** Venn diagram illustrating herb-related protein scope and comparative proximity of four herb-related proteins to PD-associated proteins. **D** Overview of three hierarchical methods for calculating herb-related protein weights

To validate the TCMNet approach, we analyzed four PD-related TCM formulas (QZS, DBYW, LWDH, TGD). Literature analysis (Fig. 2B) shows that TGD was the most frequently cited formula for PD in both CNKI and SciFinder databases, indicating high research attention and potential clinical potential. Using the BATMAN-TCM2.0 database, we identified compound-target interactions for each herb. TGD, with the most herbs and ingredients, had the broadest herb-related protein scope (5980 proteins), followed by LWDH, QZS, and DBYW (Fig. 2B).

Traditional network pharmacology approaches often consider direct target overlaps, neglecting indirect regulatory interactions mediated by PPI networks. To address this limitation, we mapped all herb-related proteins and prioritized PD-associated proteins (top-N) onto a global PPI network, quantifying therapeutic potential via proximity-based intervention strength. As shown in the line chart of Fig. 2C, TGD consistently achieved the smallest proximity values across expanding PD-associated protein sets (N = 100–1000), implying greater overall capacity to affect PD-associated targets—consistent with its high literature prevalence.

Previous studies have typically neglected the inclusion of herb-related protein weights [34–36]. We therefore implemented three hierarchical weighting strategies to refine herb-related protein prioritization. The first method considered only herb composition proportions, disregarding cumulative compound effects. The second approach expanded this by integrating herb proportions with compound abundance data. Finally, the third and most biologically comprehensive method incorporated herb proportions, compound abundance, and compound-target interaction probabilities derived from binding affinity predictions (Fig. 2D).

**Fig. 3:**
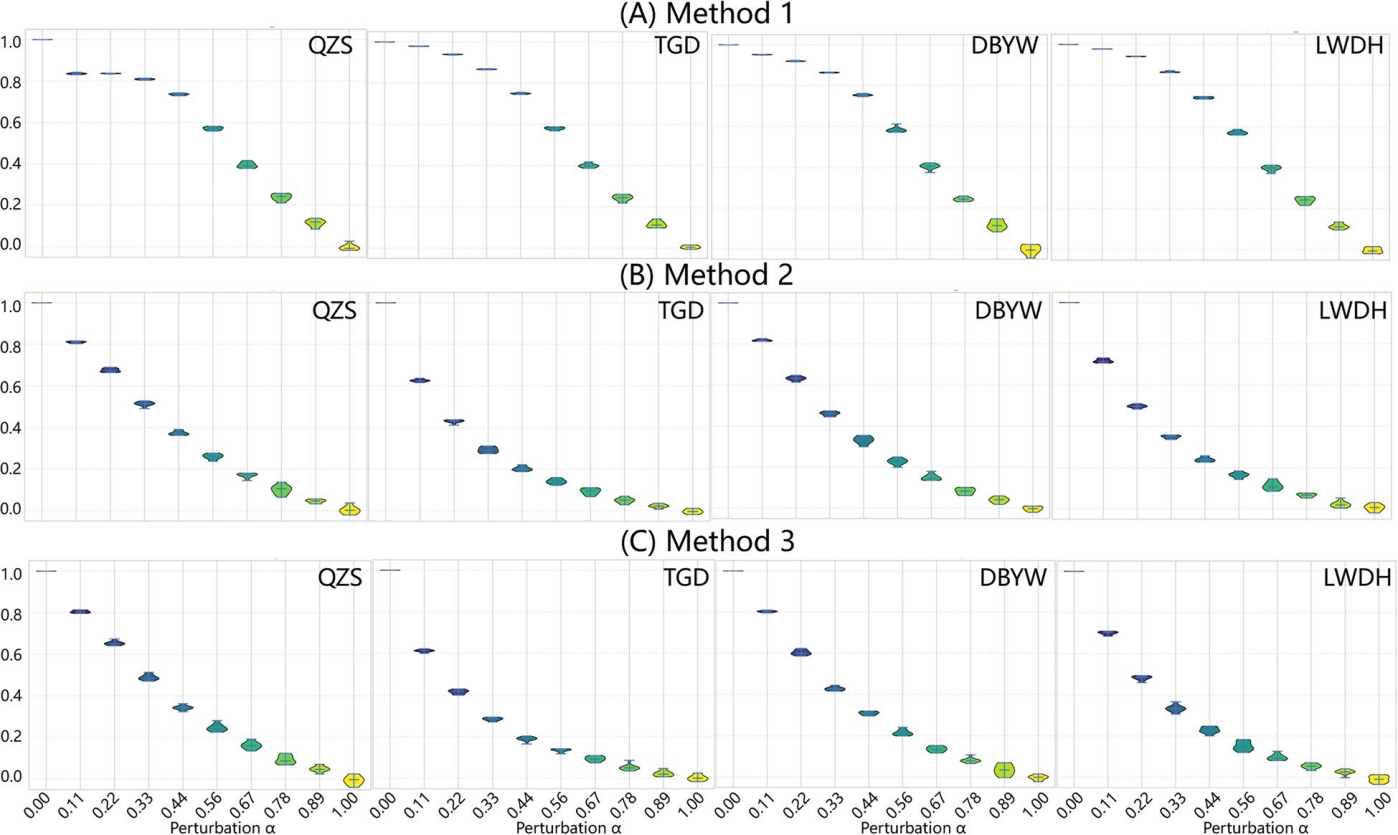
Robustness analysis (Spearman correlation, Rs) of herb-related protein weight rankings for four TCM formulas (QZS, TGD, DBYW, and LWDH) under random perturbation (α ∈ [0, 1]). The plots represent the distribution of results from 100 independent simulations performed for each α value

We evaluated the robustness of the three weighting methods using weight perturbation experiments, where the noise factor α was varied from 0 to 1. As α increased, all methods showed a gradual decline in performance, demonstrating sensitivity to weight fluctuations. Importantly, the incorporation of detailed biochemical information in the weighting schemes did not compromise the robustness of node weights; all three methods maintained comparable stability across the full perturbation range (Fig. 3). These findings suggest that, despite increased complexity, the weighted approaches remain robust and reliable in network pharmacology analyses.

We also evaluated whether high-weight herb-related proteins (Top-N, derived from each method) could effectively cover high-weight PD-associated proteins. As shown in Fig. S3, TGD achieved the highest coverage_overlap, particularly when using fewer herb-related proteins (Top100 to Top600), suggesting more efficient prioritization. This pattern was consistent across different PD-associated protein sets (Top8, Top100, Top167).

Similar trends in robustness and target coverage analyses were observed among the three weighting methods, indicating incorporating detailed biochemical data does not compromise network stability. However, Methods 1 and 2 fundamentally assume a uniform interaction probability (P = 1.0) for all identified compound-target pairs, inevitably introducing biological false positives by treating weak interactions equivalent to strong ones. In contrast, Method 3 integrates compound-target interaction probabilities as a physiological filter, explicitly down-weighting low-affinity interactions. Therefore, although all methods show statistical consistency in robustness, Method 3 was selected as the optimal strategy because it is the only model that adheres to the pharmacological principle that therapeutic efficacy depends on binding affinity. Method 3 was adopted for subsequent analyses.

### Efficacy of weighted proximity metrics

We applied three network-based metrics to comprehensively evaluate how each TCM formula targets PD. Specifically, coverage_overlap measures the direct overlap, defined as the fraction of herb-related proteins directly shared with disease-associated proteins; coverage_directlink assesses indirect regulation, indicating the fraction of herb-related proteins connected directly or through immediate neighbors to disease-associated proteins; Jaccard similarity quantifies the overall similarity between the herbal and disease-associated protein sets. As shown in Fig. S4, across all indicators, TGD again shows the strongest target engagement, followed by QZS, LWDH, and DBYW. The Jaccard similarity scores between herbal and disease targets were generally low, indicating limited direct overlap. Notably, Coverage_directlink values approached 1.0 for all formulas, reflecting dense connectivity within the PPI network and underscoring the substantial potential of TCM formulas for indirect modulation of disease-associated proteins.

**Fig. 4:**
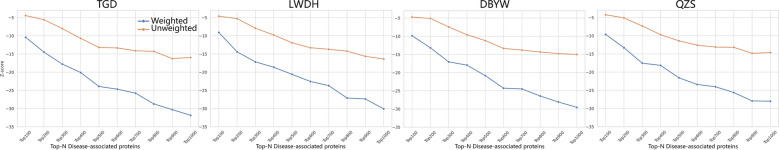
Comparison of weighted and unweighted proximity Z-scores across four TCM formulas, highlighting significant improvement from node weighting. Proximity Z-scores were calculated using the mean and standard deviation of proximity values derived from 500 random samplings for each formula. “Weighted” and “Unweighted” indicate proximity Z-scores calculated with and without node weights, respectively

To further confirm the advantage of incorporating node weights, we systematically compared weighted and unweighted network proximity across the four formulas. Additionally, to rigorously assess statistical significance, we generated null distributions by performing 500 rounds of random sampling, calculating randomized node proximity to disease-associated protein subsets, and computing corresponding Z-scores. A convergence analysis confirmed that 500 permutations are sufficient to reach stable Z-scores (Fig. S5). The results consistently showed that weighted proximity Z-scores were significantly more negative than their unweighted counterparts across all tested PD-associated protein subsets (TopN disease-associated targets) and formulas (Fig. 4). To rigorously account for the correlation between nested Top-N target subsets, we evaluated the impact of node weighting using a linear mixed-effects model. The analysis revealed a robust and statistically significant advantage for the weighted approach: weighted targets consistently yielded lower (more therapeutic) proximity Z-scores compared to unweighted ones across all formulas (Main effect: p < 1 × 10^−23^). Furthermore, this performance gap widened as more targets were included (Fig. S6).

To assess the robustness of our findings to potential biases in the PPI interactome, we repeated the proximity analysis on an independent STRING v12.0 network. Consistently, the weighted strategy maintained its superiority, producing markedly more negative proximity Z-scores than the unweighted scheme for all four formulas in this validation network (Fig. S7A). Beyond methodological robustness, we examined the consistency of therapeutic predictions. A cross-network comparison of mean proximity Z-scores revealed that the relative therapeutic rankings were preserved between the Cheng et al. and STRING networks (Rp = 0.84) (Fig. S7B). Most notably, Tianma Gouteng Decoction (TGD) consistently exhibited the lowest (most negative) weighted proximity Z-scores across both interactomes. This finding aligns closely with our literature mining results, which revealed that TGD has received the highest level of research attention and clinical validation, further supporting its therapeutic prominence. These results robustly highlight the significant enhancement in precision and reliability achieved through TCMNet’s node-weighted strategy in network pharmacology analysis, and support TGD as the highest potential TCM formula among those evaluated for PD intervention, subject to further experimental validation.

### *Case study 1*: Validation of formula optimization and integrative therapeutic prediction

To address the critical challenge of formula optimization in TCM modernization, we applied TCMNet to evaluate two optimization scenarios: (1) the evolution from classic formulas to a modern clinically optimized formula, and (2) the integrative combination of TCM with Western medicine.

**Fig. 5:**
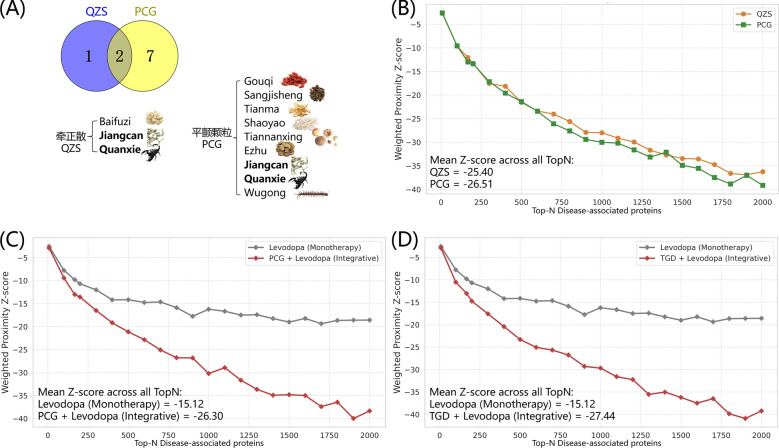
In silico validation of formula optimization and prediction of integrative therapeutic potential. **A** Venn diagram illustrating the herbal compositional relationship between the modern optimized formula Pingchan Granule (PCG) and the classic formulas, Qianzheng San (QZS). **B** Validation of formula optimization efficacy. The weighted proximity Z-scores of PCG were compared with QZS across varying Top-N PD-associated targets. **C**–**D** Evaluation of integrative optimization strategies combining TCM with Western medicine. The network proximity of Levodopa monotherapy was compared with its combination with **C** PCG and **D** TGD. Both integrative combinations (red lines) exhibited significantly stronger network proximity (lower Z-scores) compared to Levodopa alone (gray lines), aligning with clinical evidence that TCM adjunct therapy enhances efficacy. The inset text displays the mean Z-scores calculated across all Top-N thresholds

We selected Pingchan Granule (PCG), a modern clinical preparation widely used for PD [37], as a representative case. While there is no definitive historical record detailing PCG's derivation, a compositional analysis reveals that PCG shares a foundational structure with the classic Qianzheng San (QZS) [38]. As illustrated in Fig. 5A, the Venn diagram highlights that PCG retains the potent insect medicines (Scorpio and Bombyx Batryticatus) from QZS, which are crucial for extinguishing wind and stopping tremors, while incorporating additional herbs to tonify the liver and kidney. We calculated the weighted proximity Z-scores to computationally validate this formulation strategy. As shown in Fig. 5B, PCG achieved a robust mean Z-score of −26.51, which is superior to that of QZS (− 25.40). This quantitative improvement aligns with the clinical evolution of these formulas: while QZS was historically indicated for stroke-induced facial paralysis [39], the optimized PCG demonstrates a stronger, disease-specific capability to modulate the PD network. This case validates TCMNet’s ability to computationally distinguish the efficacy gains resulting from rational herbal adjustments.

Beyond formulation modification, we also explored integrative strategies. Levodopa remains the gold standard for PD treatment but is often associated with long-term side effects and efficacy fluctuations. Clinical studies have increasingly shown that combining Levodopa with TCM (e.g., PCG or TGD) can improve motor symptoms, delay dosage increases, and enhance quality of life compared to monotherapy [27, 40]. We evaluated the optimization strategy of combining TCM with Western medicine using the TCMNet. While Levodopa alone showed a moderate Z-score (− 15.12), the combinatorial regimens of PCG with Levodopa and TGD with Levodopa achieved significantly enhanced Z-scores (− 26.30 and − 27.44, respectively) (Fig. 5C, D). These findings indicate that the multi-target network regulation of TCM effectively complements the specific dopaminergic targeting of Levodopa, providing compelling in silico support for the observed clinical advantages of integrative therapy.

### *Case study 2*: Identification of bioactive constituents in single-herb medicine

To further demonstrate the utility of the TCMNet framework beyond classical multi-herb formulas, we selected *Ginkgo biloba*, a widely studied single-herb medicine with neuroprotective effects [41, 42], as a representative case to evaluate its network-based therapeutic potential against PD.

**Fig. 6:**
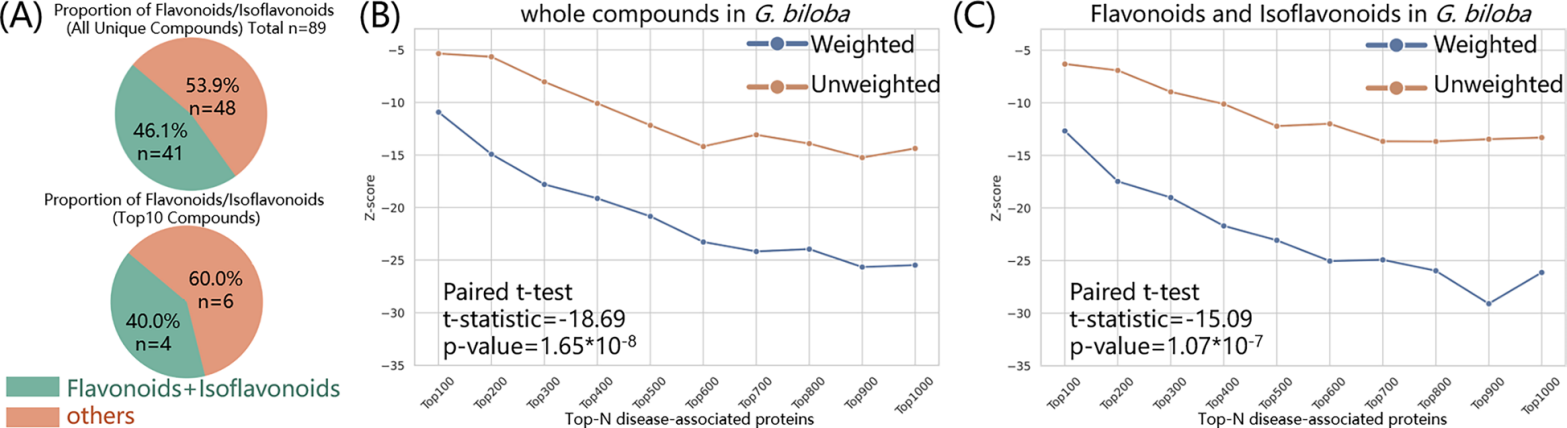
Network-based pharmacological analysis of *Ginkgo biloba* compounds against Parkinson’s disease (PD). **A** Proportion of flavonoids and isoflavonoids among all identified compounds (top) and among the top 10 most abundant compounds (bottom) in *Ginkgo biloba*. **B** Z-scores of network proximity between the full *Ginkgo biloba-related* protein set and PD-associated proteins across varying top-N subsets. **C** Z-scores of proximity between the flavonoid/isoflavonoid-derived proteins and PD-associated proteins

Using the proprietary Meta-TCM database, small molecules from *Ginkgo biloba* were identified along with their relative abundances. After filtering and weighting the compound-protein pairs based on both compound abundance and predicted interaction probabilities, we constructed a weighted herb-related protein profile comprising 1585 proteins regulated by all compounds in *Ginkgo biloba*. Further structural classification of the constituent compounds revealed dozens of chemotypes, among which flavonoids and isoflavonoids accounted for 46% of the total (Fig. 6A, top). Notably, 40% of the top 10 most abundant compounds (Fig. S8) belonged to these two classes (Fig. 6A, bottom), collectively targeting 650 unique proteins.

We then evaluated the ability of the full herb-related protein set and the flavonoid/isoflavonoid-related protein subset to modulate PD-related proteins by computing proximity metrics and associated Z-scores based on 500 rounds of degree-matched random sampling. As shown in Fig. 6B and C, the flavonoid subset consistently exhibited lower Z-scores than the full compound set, suggesting stronger topological association with PD-related proteins. These results confirm that incorporating node weights significantly enhances the predictive power of proximity-based analyses, enabling the model to more sensitively capture biologically relevant interactions between critical nodes.

**Fig. 7:**
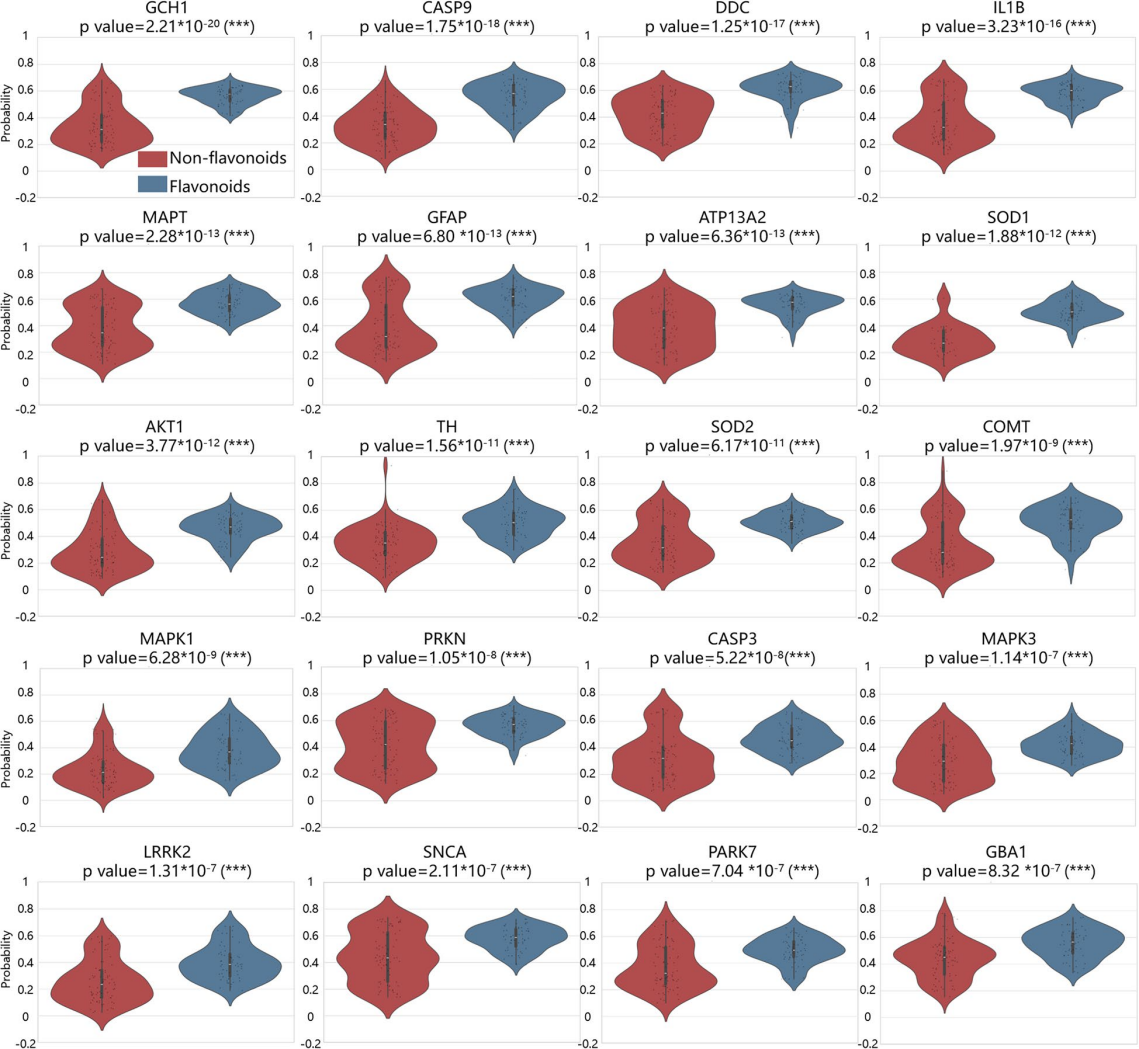
Violin plots comparing the predicted binding probabilities of flavonoid (blue) and non-flavonoid (red) compounds from *Ginkgo biloba* against the 20 high-priority PD-associated proteins. The y-axis shows the predicted probability of ligand–protein interaction (range: 0–1) calculated by the Boltz-2 model; higher values indicate a greater likelihood of binding. Statistical significance between groups was evaluated using the independent two-sample Welch’s t-test. Significance is indicated as follows: ns (p > 0.05), *(p ≤ 0.05), **(p ≤ 0.01), ***(p ≤ 0.001). In each violin plot, the white dot represents the median, and the thick black bar indicates the interquartile range (25–75th percentile). “Flavonoids” refers to both flavonoids and isoflavonoids (shown in blue), while “Non-Flavonoids” are shown in red

**Fig. 8:**
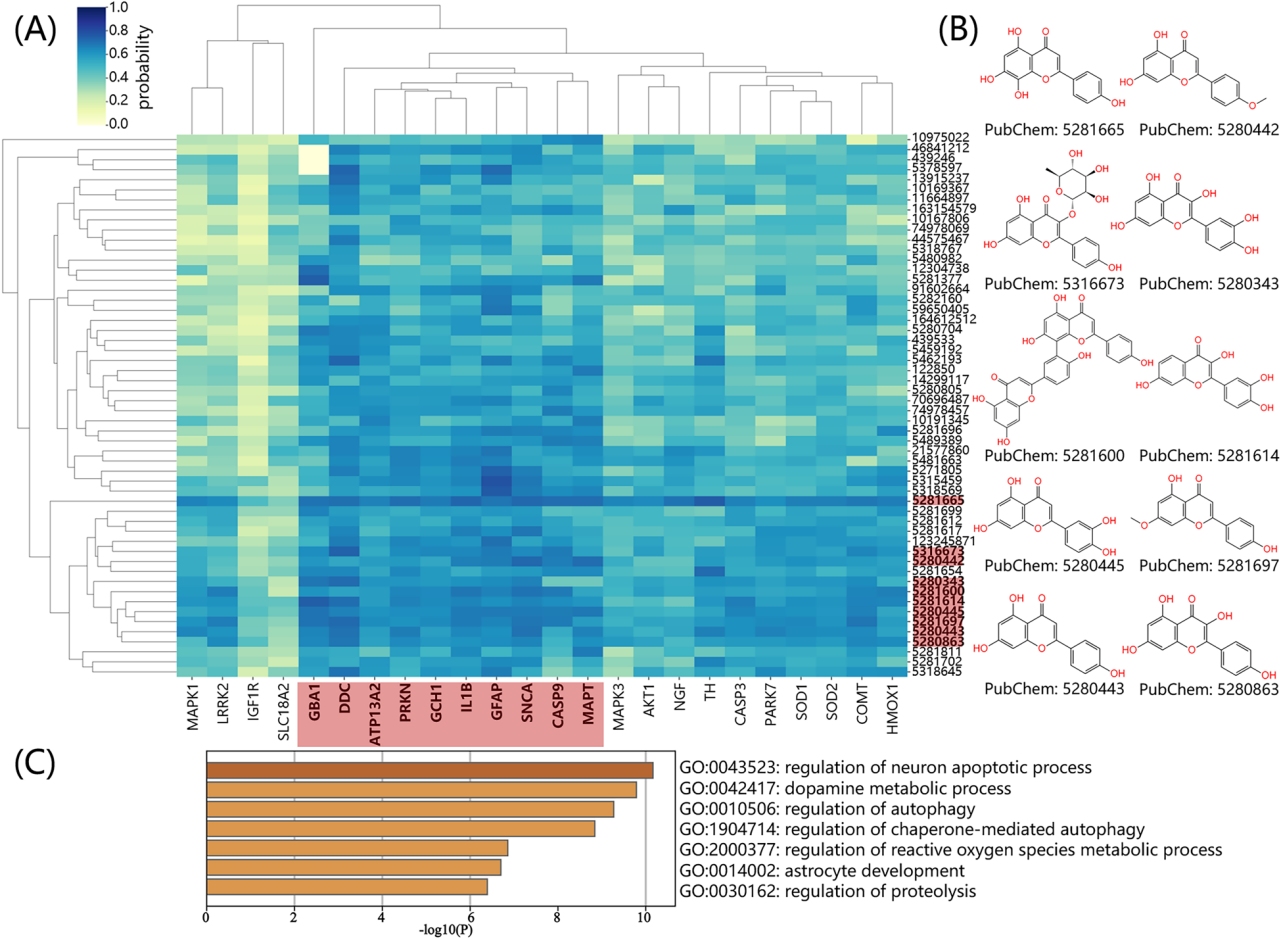
Hierarchical clustering analysis of flavonoid–protein interaction probabilities in Parkinson’s disease. **A** Heatmap showing the predicted binding probability matrix (range: 0–1, color-coded) between all flavonoid compounds from *Ginkgo biloba* and 24 high-priority PD-associated proteins. Both rows (compounds) and columns (proteins) are clustered by similarity. **B** Representative chemical structures of Isoscutellarein and its analogs (highlighted in red in Fig.  8 A), identified as preferential binders to key PD proteins. These compounds were selected based on their high mean binding probabilities across the panel of 30 proteins, indicating their broad-spectrum binding potential. **C** Functional enrichment analysis of the protein clusters targeted by flavonoids, with Gene Ontology terms related to neuronal apoptosis, dopamine metabolism, autophagy, astrocyte development, and proteolysis (enrichment significance indicated by log10(p)). This analysis highlights the functional clustering of PD-associated proteins most susceptible to flavonoid modulation

To further illustrate the TCMNet’s ability to identify key bioactive constituents and clarify the molecular mechanisms underlying the anti-Parkinson’s activity of *Ginkgo biloba*, we systematically compared the predicted binding probabilities of flavonoid and non-flavonoid compounds against a panel of 30 high priority PD-associated proteins using the Boltz-2 deep learning model (Fig. 7, Fig. S9). Binding probabilities were compared using an independent two-sample Welch’s *t*-test for each protein. The 20 proteins showing the largest significant increase in binding probability for flavonoids are presented in Fig. 7; the remaining 10 proteins are shown in Fig. S9. Overall, flavonoids exhibited significantly higher predicted interaction probabilities for 24 of the 30 proteins (p < 0.001), supporting TCMNet’s AI-driven predictions that flavonoids preferentially target key PD-associated proteins. Furthermore, a hierarchical clustering heatmap (Fig. 8A) was used to visualize the predicted interaction probability matrix between all flavonoid compounds and the 24 high-confidence PD-associated protein targets. The clustering results revealed that certain flavonoids, such as Isoscutellarein and its analogs (Fig. 8B), preferentially target clusters of proteins involved in neuronal apoptosis, dopamine metabolism, autophagy, and astrocyte development (Fig. 8C).

Together, these findings illustrate a distinct therapeutic mode: unlike the highly selective inhibitors designed for single PD-associated protein (e.g., LRRK2) [43, 44], flavonoids in *Ginkgo biloba* exhibit a multi-target binding profile. Consequently, they represent the major bioactive chemical classes in *Ginkgo biloba* contributing to PD intervention. More importantly, they demonstrate that integrating node weight into proximity-based models substantially enhances the predictive power of network pharmacology analyses. TCMNet thus not only enables the quantification of target engagement at the systems level but also facilitates the identification of key active constituents, providing a rational basis for herbal formula simplification and optimization.

## Discussion

Our study proposes TCMNet, an advanced AI network strategy integrating node weight to systematically optimize and simplify traditional Chinese medicine (TCM) formulas. Traditional network pharmacology [45–47] approaches often rely solely on overlaps of herb-related proteins, neglecting protein node relevance and indirect regulatory interactions within protein–protein interaction (PPI) networks. TCMNet addresses this critical gap by explicitly weighting protein nodes based on disease relevance and herbal composition characteristics, thereby substantially improving the biological accuracy and interpretability of predicted therapeutic outcomes.

Application of TCMNet to four classical PD-relevant formulas consistently revealed improved predictive performance upon integration of node weights. Notably, Tianma Gouteng Decoction exhibited robust therapeutic potential, corresponding with its documented clinical efficacy. Additionally, our comprehensive case study of *Ginkgo biloba* further illustrated the advantages of TCMNet. Flavonoids and isoflavonoids represented nearly half of the total compounds in *Ginkgo biloba*. TCMNet identified them as the predominant active constituents, aligning well with their known neuroprotective properties [48]. Validated by state-of-the-art deep learning predictions, our analysis further revealed that these compounds possess strong binding affinities for multiple key proteins associated with PD. While computational, the biological relevance of this finding is substantiated by robust epidemiological evidence: large-scale prospective studies involving over 120,000 participants have linked habitual flavonoid intake to a reduced risk of developing PD [49], while higher consumption correlates with lower mortality rates in diagnosed patients [50]. This concordance between our in silico method and real-world clinical outcomes underscores TCMNet’s capacity to capture biologically meaningful therapeutic signals. Importantly, node weighting markedly enhanced the resolution of network proximity metrics, affirming the effectiveness of our approach in distinguishing core therapeutic constituents.

This methodology holds considerable promise for modernizing TCM research, offering an evidence-based approach for formula optimization, active ingredient identification, and mechanistic elucidation. Despite the demonstrated strengths, our study has limitations. First, node weight accuracy depends heavily on the underlying data quality, which could introduce biases from incomplete or heterogeneous databases. Current public literature may suffer from “study bias,” where well-investigated proteins have more documented interactions. This imbalance may skew the network topology, potentially leading the model to overestimate the therapeutic proximity of herbs targeting these “popular” proteins simply due to their high connectivity. While our weighting strategy partially mitigates this by integrating multi-source data, these data-intrinsic limitations imply that our rankings should be interpreted as prioritized hypotheses rather than definitive confirmations. Furthermore, to mitigate inherent AI risks such as hallucinations or misinterpretations, we integrated LLM outputs with objective evidence from authoritative databases, ensuring that high-priority targets are supported by robust multi-source consensus. We emphasize that TCMNet currently functions as an evaluative algorithm to screen interactions, rather than a generative model for creating new herb combinations. Consequently, the present validation is retrospective, relying on established clinical formulas. To address this limitation, future research should include prospective application of TCMNet for novel formula optimization, followed by rigorous experimental validation at cellular and organismal levels. Moreover, incorporating advanced multimodal deep learning techniques, such as deep graph neural networks and multi-omics integration, represents a promising direction. These approaches could enhance the identification and clustering of biologically meaningful nodes, thereby refining disease module definitions and therapeutic target predictions within the network pharmacology framework.

## Conclusions

In conclusion, the TCMNet strategy provides a robust and biologically meaningful computational framework for evaluating, optimizing, and simplifying TCM formulations for complex diseases. The explicit incorporation of protein node weights significantly enhances predictive accuracy, enabling systematic identification of key bioactive constituents and their target engagement mechanisms. This strategy and our findings offer valuable insights and practical tools for the rational modernization and standardization of traditional herbal medicine research and application.

## Supplementary Information

Supplementary Material 1.

## Data Availability

All data generated or analysed during this study are included in this published article and its supplementary information files. The codes for network metric calculations and herb-related protein weighted computation are openly available at https://github.com/ZJUFanLab/TCMNet.

## Abbreviations

AI: Artificial intelligence
BFS: Breadth-First Search
CNKI: China national knowledge infrastructure
CTD: Comparative Toxicogenomics Database
DBYW: Dabuyin Wan
LLM: Large language model
LWDH: Liuwei Dihuang
PD: Parkinson’s disease
PPI: Protein protein interactions
QZS: Qianzheng San
Rs: Spearman rank correlation coefficient
SMILES: Simplified molecular input line entry system
TCM: Traditional Chinese medicine
TGD: Tianma Gouteng Decoction
TTD: Therapeutic Target Database
