# Supplementary material for: TCMNet: an AI-driven strategy for optimizing traditional Chinese medicine
Source: Chin Med. 2026 Mar 31;21:106. doi: 10.1186/s13020-026-01360-w (PMC13037211; doi:10.1186/s13020-026-01360-w)
Supplement: Supplementary file 1 — Supplementary Material 1. [file 13020_2026_1360_MOESM1_ESM.docx]

Supporting information

**TCMNet: An AI-driven strategy for optimizing traditional Chinese medicine**

Shuoyan Tan^a, b, †^, Xin Shao ^a, b, † *^, Xuting Zhang^b^, Yizheng Dai^a, b^, Boli Zhang ^c*^, Yiyu Cheng ^b*^, Xiaohui Fan ^a, b*^

^a^ State Key Laboratory of Chinese Medicine Modernization, Pharmaceutical Informatics Institute, College of Pharmaceutical Sciences, Zhejiang University, Hangzhou 310058, China.

^b^ State Key Laboratory of Chinese Medicine Modernization, Innovation Center of Yangtze River Delta, Zhejiang University, Jiaxing 314100, China.

^c^ State Key Laboratory of Chinese Medicine Modernization, Tianjin University of Traditional Chinese Medicine, Tianjin 301617, China

^†^ These authors contributed equally to this work.

^*^Corresponding authors


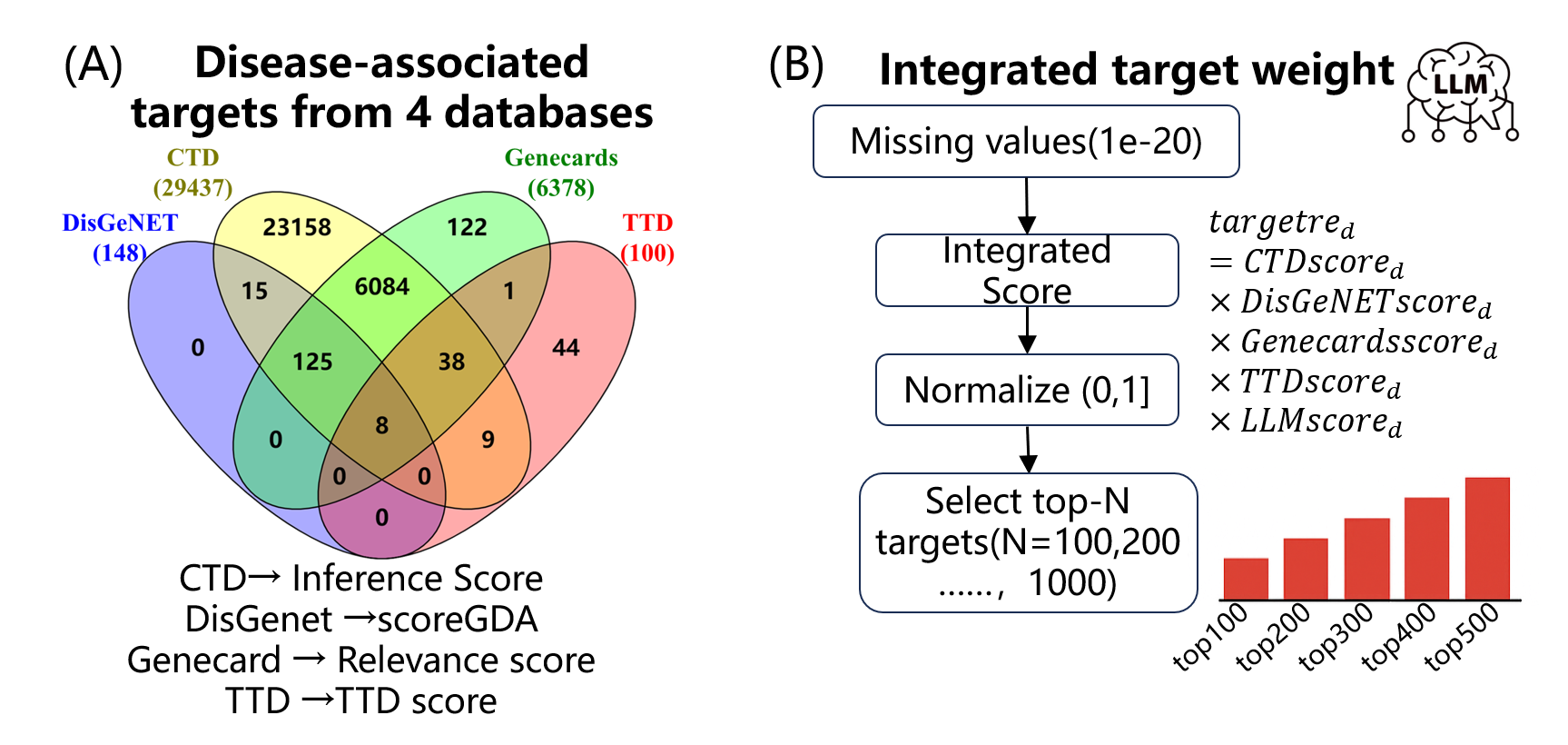


**Figure S1.** (A) Venn diagram illustrating overlaps among PD-related targets obtained from CTD, GeneCards, DisGeNET, and TTD databases. (B) Workflow for integration and normalization of disease-associated protein weights, including missing-value imputation, combined scoring, and top-N-related protein selection.


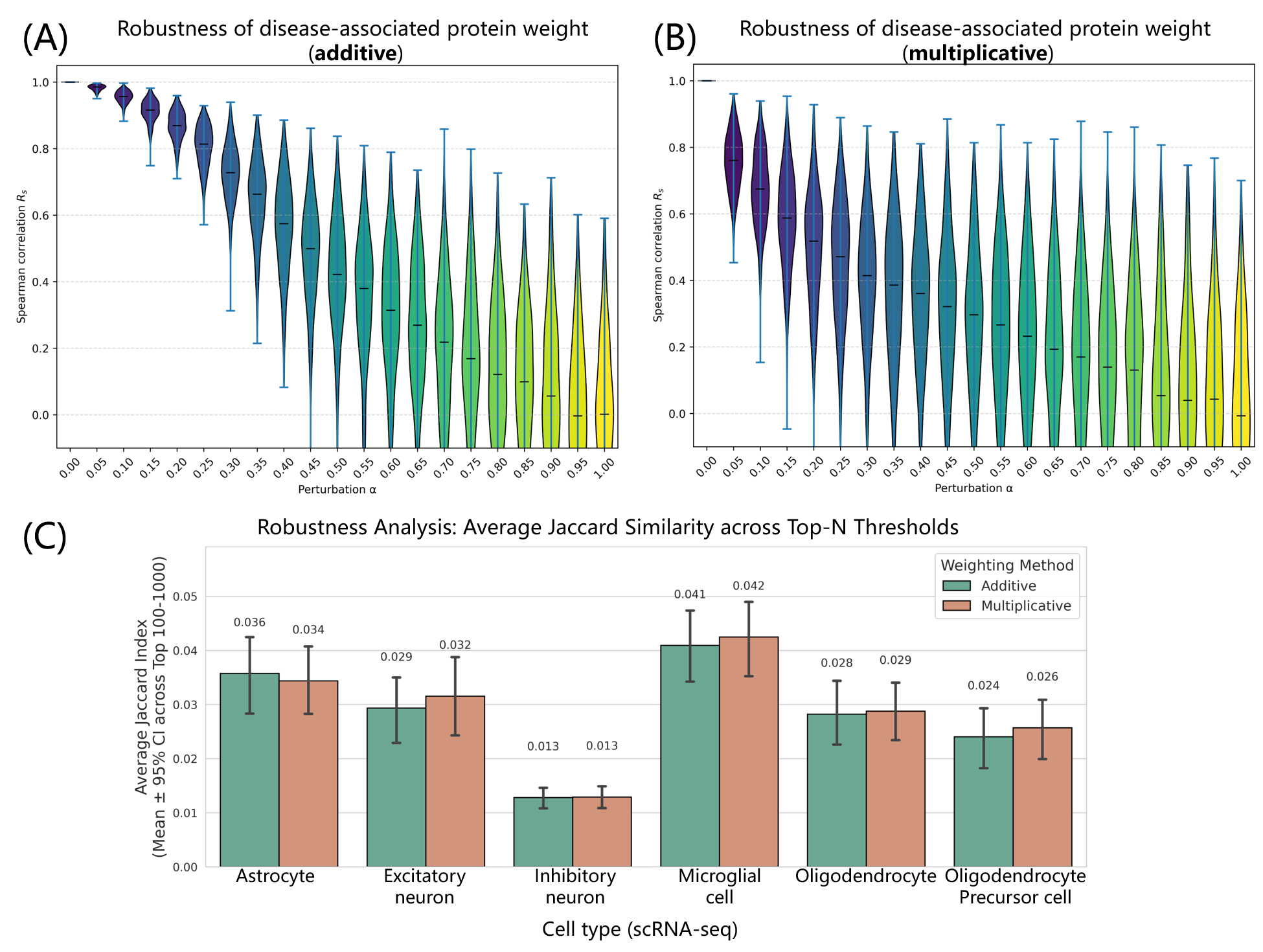


**Figure S2.** Sensitivity analysis and biological validation comparing multiplicative and additive disease-associated protein integration schemes. (A–B) Robustness of disease-associated protein rankings under random weight perturbation. Violin plots display the distribution of Spearman rank correlation coefficients (Rs) between original and perturbed rankings across varying noise levels (α ∈ [0,1]) for the (A) additive and (B) multiplicative integration methods. (C) External biological validation against single-cell transcriptomic data. The bar chart compares the average Jaccard similarity between computational disease-associated proteins (prioritized by additive vs. multiplicative schemes) and differentially expressed genes (DEGs) identified from independent scRNA-seq data (GSE202210) across six key cell types. Data represents the mean Jaccard index±95% confidence interval (CI) aggregated across Top 100–1000 protein thresholds. The multiplicative scheme (orange) demonstrates consistent or superior alignment with biological signatures, particularly in microglial cells.


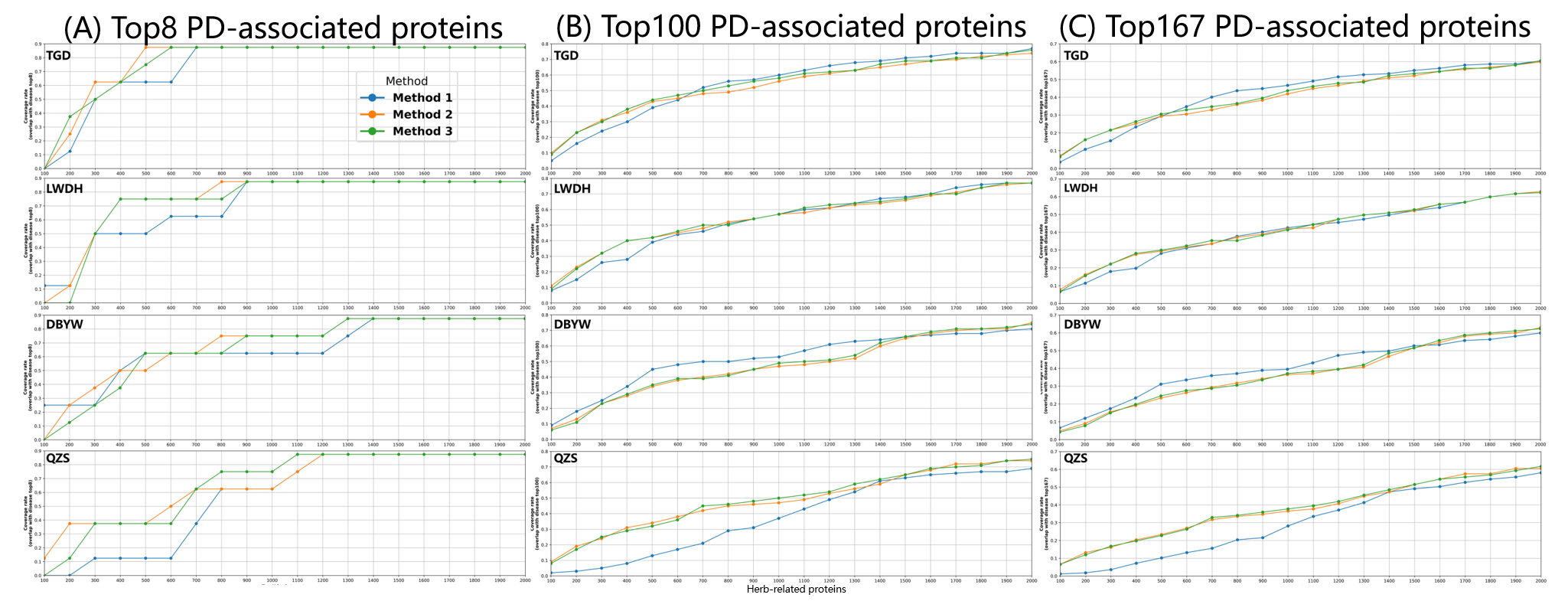


**Figure S3.** Target coverage analysis of prioritized herb-related protein (Top-N, ranked by weight) from four classical TCM formulas against top-ranked PD-associated protein targets (Top 8, Top 100, Top 167).


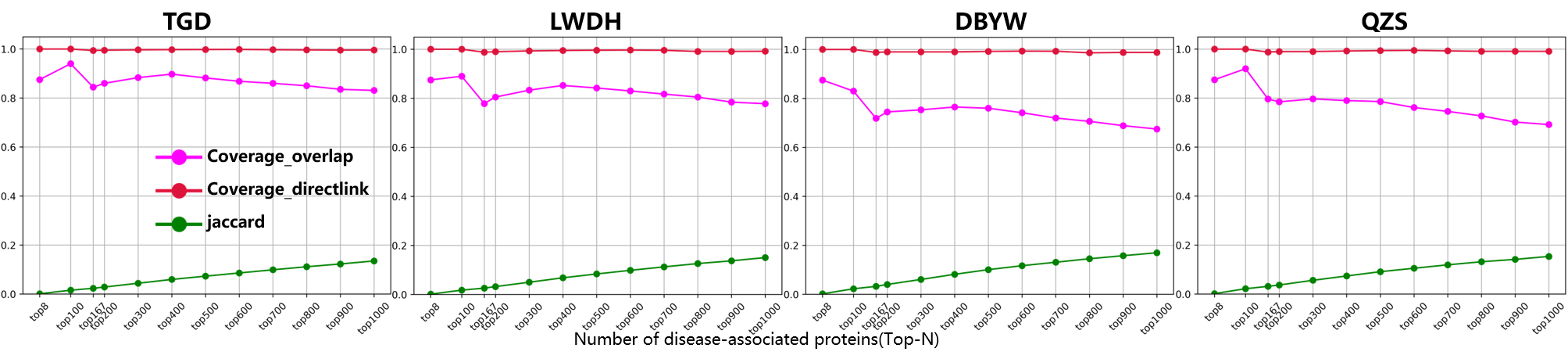


**Figure S4**. Comparative evaluation of target coverage for four TCM formulas across multiple PD-associated protein subsets, as measured by coverage_overlap (direct overlap), coverage_directlink (indirect network connectivity), and Jaccard index (set similarity).


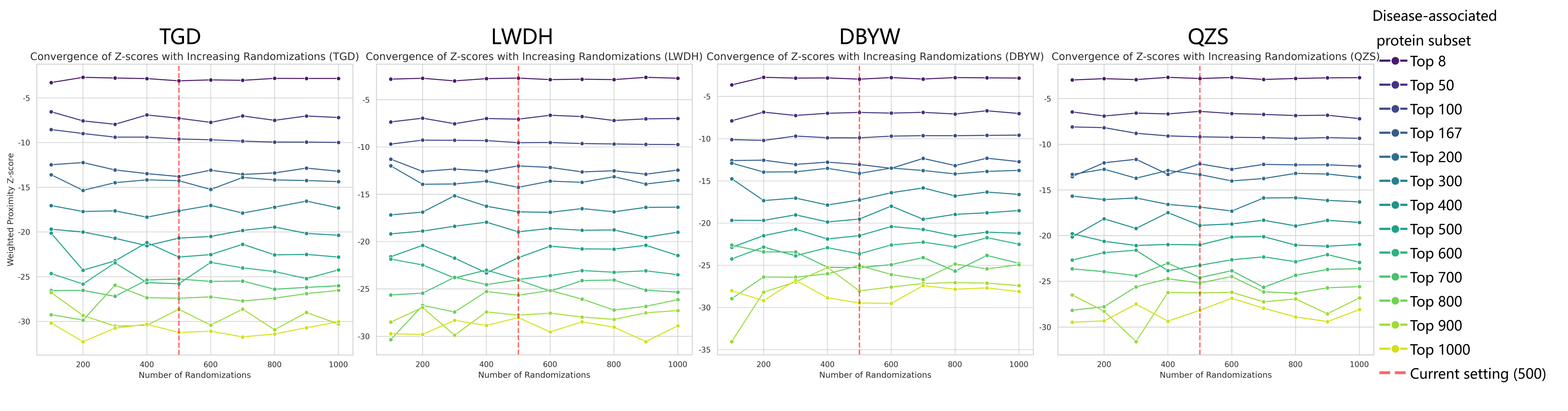


**Figure S5**. Convergence analysis of weighted proximity Z-scores across varying numbers of randomizations. The plots illustrate the stability of weighted proximity Z-scores for four TCM formulas (TGD, LWDH, DBYW, QZS) as the number of random sampling iterations increases from 100 to 1000. The x-axis represents the number of randomizations performed to generate the null distribution, and the y-axis shows the resulting weighted proximity Z-score. Different colored lines correspond to different subsets of prioritized PD-associated proteins (ranging from Top 8 to Top 1000). The vertical red dashed line indicates the threshold of 500 iterations used in the main study. The trajectories demonstrate that Z-scores stabilize rapidly, with negligible fluctuations in values (Average CV ≈ 2.5%) observed between 500 and 1000 iterations, confirming that 500 randomizations provide a statistically robust estimate while maintaining computational efficiency.


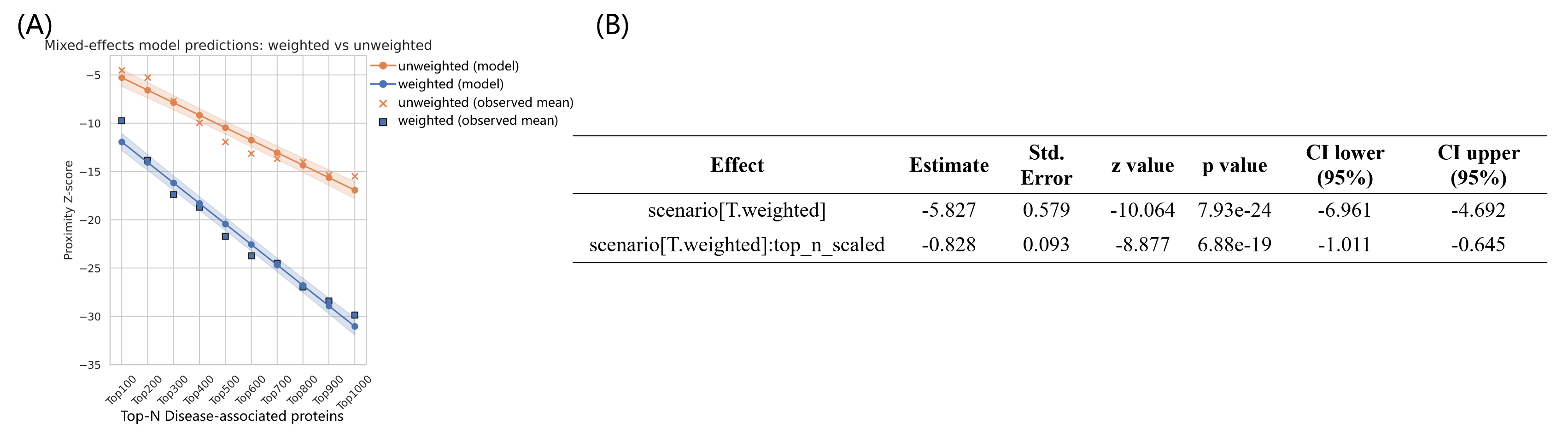


**Figure S6.** Mixed-effects modeling of weighted vs. unweighted proximity Z-scores across Top-N disease**-**associated proteins. (A) Model-based predictions of proximity Z-scores for weighted and unweighted protein schemes across Top100–Top1000 disease**-**associated proteins. Solid lines show the fixed-effects predictions from the linear mixed-effects model with Scenario (weighted vs. unweighted), scaled Top-N, and their interaction as fixed effects and a random intercept for formula (Four formulas); shaded bands indicate 95% confidence intervals. Markers represent the observed mean Z-scores across the four formulas at each Top-N threshold. (B) Fixed-effects estimates from the mixed-effects model, including the intercept, the main effects of Scenario and Top-N, and their interaction, with corresponding standard errors, z values, p values, and 95% confidence intervals.


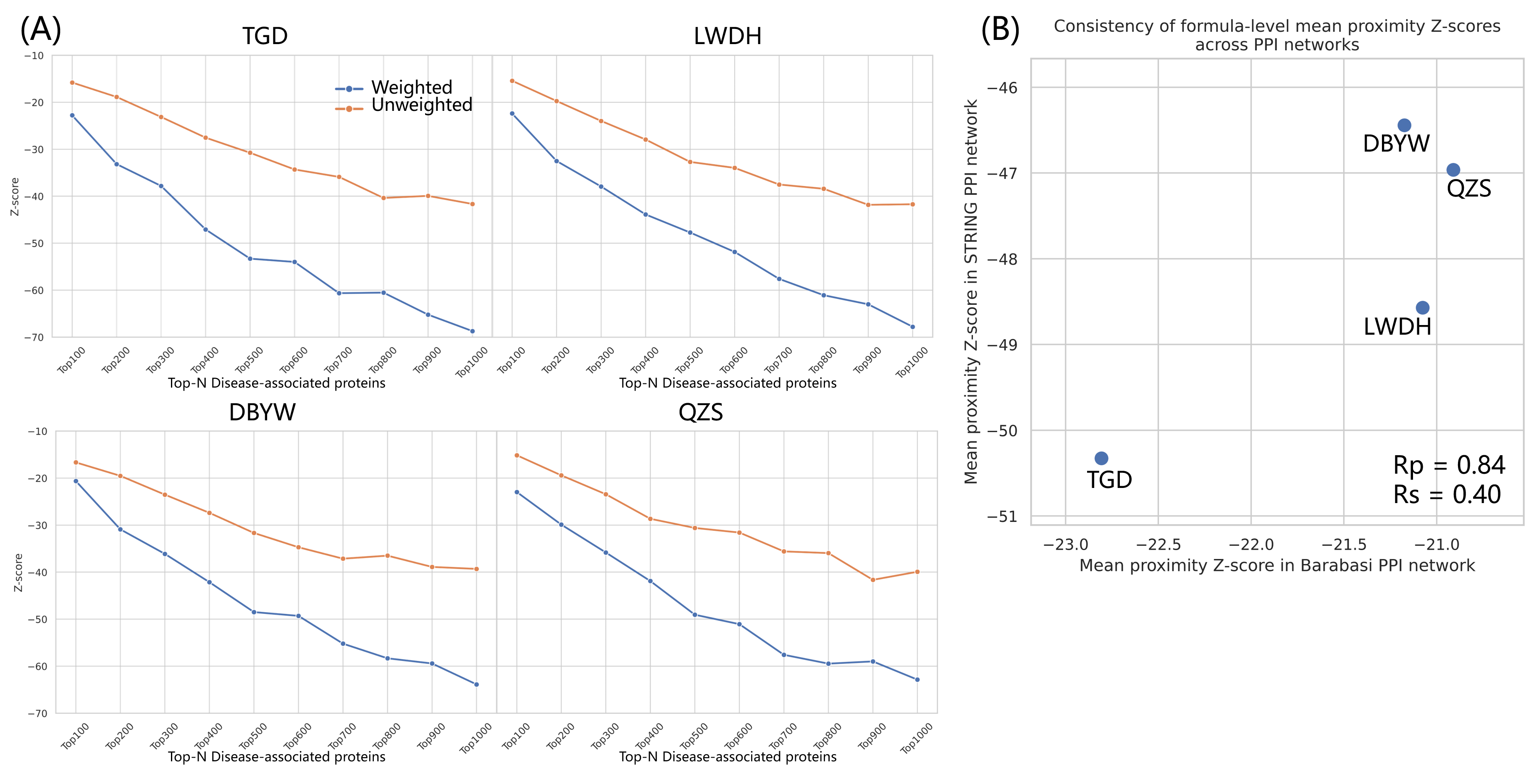


**Figure S7.** Robustness validation of TCMNet strategy using an independent STRING PPI network. To verify that the results are not dependent on a specific network dataset, we repeated the analysis using the high-confidence human PPI network from STRING v12.0 (combined_score ≥ 700). (A) Comparison of weighted and unweighted proximity Z-scores for four TCM formulas in the STRING network. The Z-scores (y-axis) were calculated against varying subsets of prioritized PD-associated proteins (Top100 to Top1000, x-axis) based on 500 random permutations. (B) Scatter plot comparing the mean proximity Z-scores of the four formulas between the two networks. Each point represents the average Z-score of a formula across all Top-N disease**-**associated protein sets. The high Pearson correlation (Rp = 0.84) and the position of TGD (bottom-left corner) demonstrate that TGD is robustly identified as the most potent formula across both independent interactomes. (Rp: Pearson correlation coefficient; Rs: Spearman rank correlation coefficient.)


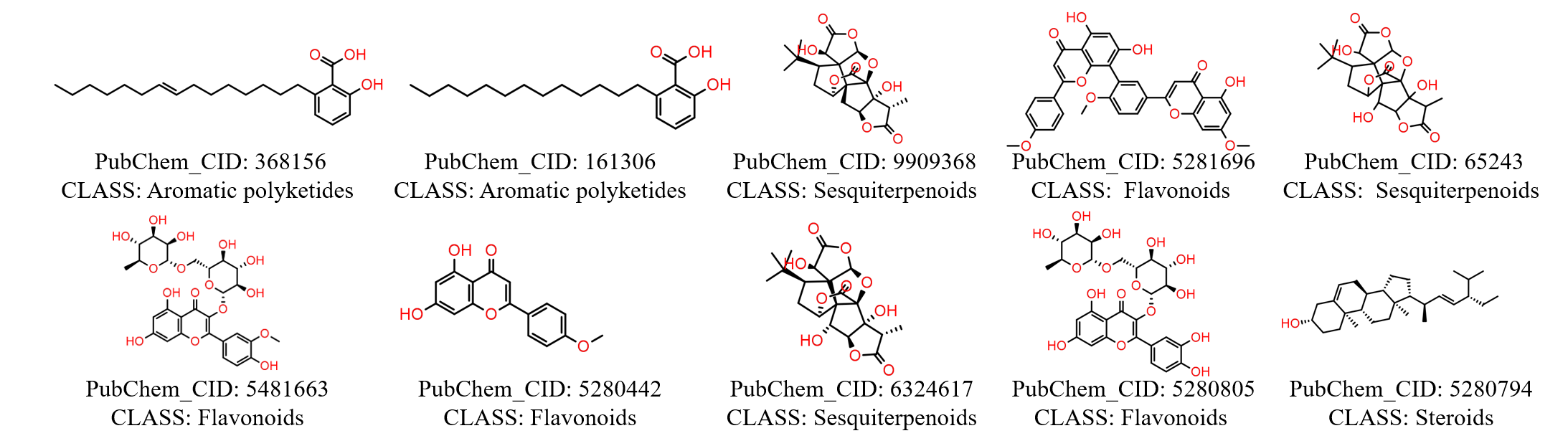


**Figure S8.** Top 10 most abundant compounds in *Ginkgo bilob*a and their corresponding structural classes, as determined from quantitative profiling of herbal constituents.


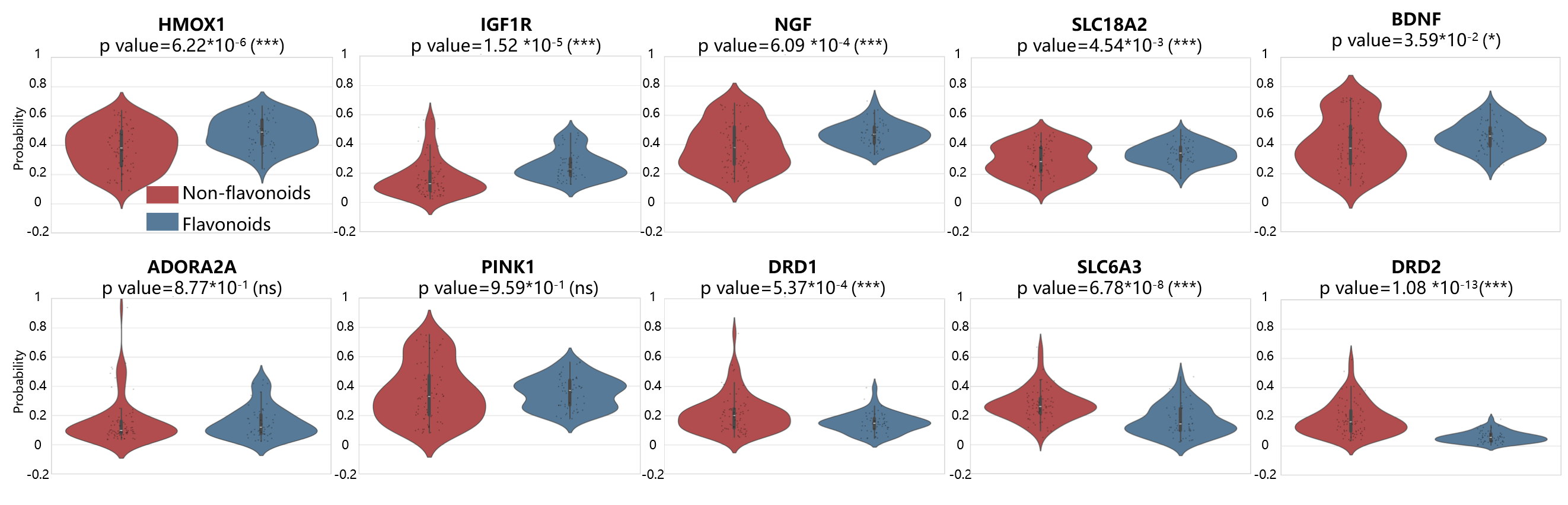


**Figure S9.** Violin plots comparing the predicted binding probabilities of flavonoid (blue) and non-flavonoid (red) compounds from *Ginkgo biloba* against the remaining 10 high-priority PD-associated protein targets. The y-axis shows the predicted probability of ligand-protein interaction (range: 0 to 1) calculated by the Boltz-2 model; higher values indicate a greater likelihood of binding.

**Table S1.** Quantitative evaluation of entity extraction performance across different models on the TCM-NER benchmark dataset.

| Model | Precision | Recall | F1-score |
| --- | --- | --- | --- |
| BERT-CRF | 0.994 | 0.807 | 0.885 |
| Qwen1.5-7B-Chat | 0.988 | 0.749 | 0.829 |
| GPT-3.5-turbo | 0.988 | 0.876 | 0.914 |
| HuatuoGPT | 0.950 | 0.662 | 0.743 |
| TCMChat1.0 | 0.975 | 0.861 | 0.907 |
| TCMChat1.5 | 0.977 | 0.868 | 0.910 |

TCM-NER benchmark dataset was downloaded from https://tianchi.aliyun.com/dataset/86819.

Bold text indicates the specific model (TCMChat1.5) adopted in the TCMNet strategy for extracting disease**-**associated proteins from literature.

The specific prompt employed for this extraction task was "Please identify all TCM herb names, disease/symptom names, and treatment plan entities from the following TCM text. "

**Table S2**. Comparison between multiplicative and additive integration of disease-associated protein.

| Top-N | Jaccard index^a^ | Spearman ρ (intersection)^b^ |
| --- | --- | --- |
| 50 | 0.64 | 0.93 |
| 100 | 0.52 | 0.86 |
| 150 | 0.75 | 0.68 |
| 200 | 0.90 | 0.64 |
| 250 | 0.81 | 0.77 |
| 300 | 0.80 | 0.84 |
| 350 | 0.76 | 0.88 |
| 400 | 0.75 | 0.91 |
| 450 | 0.75 | 0.92 |
| 500 | 0.74 | 0.93 |

a For each Top-N set of disease-associated proteins, the Jaccard index was computed to quantify the overlap between targets selected under the multiplicative versus additive integration schemes.

b For each Top-N, Spearman’s rank correlation coefficient (ρ) was calculated between the normalized scores from the multiplicative and additive integrations, restricted to the intersection of proteins identified by both schemes, to assess the consistency of protein ranking.

**Table S3**. Composition and Dosage Ratios of Constituent Herbs in Four Classic TCM Formulas for Parkinson’s Disease (PD)

| Formula Name | Herbal Name (Pinyin) | Herbal Name (Latin) | Proportion | Percentage |
| --- | --- | --- | --- | --- |
| 牵正散  (QZS) | 白附子 (Bai Fu Zi) | Typhonii Rhizoma | 1 | 0.33 |
|  | 僵蚕 (Jiang Can) | Bombyx Batryticatus | 1 | 0.33 |
|  | 全蝎 (Quan Xie) | Scorpio | 1 | 0.33 |
| 大补阴丸  (DBYW) | 熟地黄 (Shu Di Huang) | Rehmanniae Radix Praeparata | 3 | 0.30 |
|  | 龟板 (Gui Ban) | Carapax Testudinis | 3 | 0.30 |
|  | 黄柏 (Huang Bai) | Phellodendri Chinensis Cortex | 2 | 0.20 |
|  | 知母 (Zhi Mu) | Anemarrhenae Rhizoma | 2 | 0.20 |
| 六味地黄  (LWDH) | 地黄 (Di Huang) | Rehmanniae Radix | 8 | 0.27 |
|  | 山茱萸 (Shan Zhu Yu) | Cornus Officinalis Sieb. Et Zucc. | 4 | 0.13 |
|  | 山药 (Shan Yao) | Rhizoma Dioscoreae | 4 | 0.13 |
|  | 茯苓 (Fu Ling) | Poria | 3 | 0.10 |
|  | 牡丹皮 (Mu Dan Pi) | Cortex Moutan | 3 | 0.10 |
|  | 泽泻 (Ze Xie) | Alismatis Rhizoma | 3 | 0.10 |
| 天麻钩藤汤  (TGD) | 天麻 (Tian Ma) | Gastrodiae Rhizoma | 3 | 0.09 |
|  | 钩藤 (Gou Teng) | Uncariae Ramulus Cumuncis | 4 | 0.12 |
|  | 石决明 (Shi Jue Ming) | Haliotidis Concha | 6 | 0.18 |
|  | 栀子 (Zhi Zi) | Gardeniae Fructus | 3 | 0.09 |
|  | 黄芩 (Huang Qin) | Scutellariae Radix | 3 | 0.09 |
|  | 川牛膝 (Chuan Niu Xi) | Cyathulae Radix | 4 | 0.12 |
|  | 杜仲 (Du Zhong) | Eucommiae Cortex | 3 | 0.09 |
|  | 益母草 (Yi Mu Cao) | Leonuri Herba | 3 | 0.09 |
|  | 桑寄生 (Sang Ji Sheng) | Herba Taxilli | 3 | 0.09 |
|  | 夜交藤 (Ye Jiao Teng) | Caulis Polygoni Multiflori | 3 | 0.09 |
|  | 茯苓 (Fuling) | Poria | 3 | 0.09 |
| 平颤颗粒Pingchan granule (PCG) | 枸杞 (Gou Qi) | Lycii Fructus | 4 | 0.13 |
|  | 桑寄生 (Sang Ji Sheng) | Herba Taxilli | 5 | 0.17 |
|  | 天麻 (Tian Ma) | Gastrodiae Rhizoma | 3 | 0.10 |
|  | 芍药 (Shao Yao) | Paeoniae Alba Radix | 5 | 0.17 |
|  | 天南星 (Tian Nan Xing) | Arisaematis Rhizoma | 5 | 0.17 |
|  | 莪术 (E Zhu) | Curcumae Radix | 3 | 0.10 |
|  | 僵蚕 (Jiang Can) | Bombyx Batryticatus | 3 | 0.10 |
|  | 全蝎 (Quan Xie) | Scorpio | 1 | 0.03 |
|  | 蜈蚣(Wu Gong) | Scolopendra | 1 | 0.03 |

**Table S4**. Top 30 PD-associated protein targets and their integrated weights, as determined by multi-databases scoring.

| ID | GeneSymbol | Uniport ID | source | retotalscore |
| --- | --- | --- | --- | --- |
| 1 | SNCA | P37840 | CTD; DisGeNET; GeneCards; TTD; LLM | 1 |
| 2 | TH | P07101 | CTD; DisGeNET; GeneCards; TTD; LLM | 0.17473944 |
| 3 | LRRK2 | Q5S007 | CTD; DisGeNET; GeneCards; TTD; LLM | 0.084641883 |
| 4 | DDC | P20711 | CTD; DisGeNET; GeneCards; TTD; LLM | 0.071471222 |
| 5 | MAPT | P10636 | CTD; DisGeNET; GeneCards; TTD; LLM | 0.040949716 |
| 6 | AKT1 | P31749 | CTD; DisGeNET; GeneCards; TTD; LLM | 0.016729897 |
| 7 | GBA1 | P04062 | CTD; DisGeNET; GeneCards; TTD; LLM | 0.007277935 |
| 8 | IGF1R | P08069 | CTD; DisGeNET;GeneCards; TTD; LLM | 0.000660325 |
| 9 | PRKN | O60260 | CTD; DisGeNET; GeneCards; LLM | 4.90E-21 |
| 10 | PINK1 | Q9BXM7 | CTD; DisGeNET; GeneCards; LLM | 3.55E-21 |
| 11 | PARK7 | Q99497 | CTD; DisGeNET; GeneCards; LLM | 3.17E-21 |
| 12 | SLC6A3 | Q01959 | CTD; DisGeNET; GeneCards; LLM | 1.19E-21 |
| 13 | COMT | P21964 | CTD; GeneCards; TTD; LLM | 9.27E-22 |
| 14 | SOD1 | P00441 | CTD; DisGeNET; GeneCards; LLM | 8.17E-22 |
| 15 | DRD2 | P14416 | CTD; DisGeNET; GeneCards; LLM | 8.15E-22 |
| 16 | GCH1 | P30793 | CTD; GeneCards; TTD; LLM | 6.39E-22 |
| 17 | SOD2 | P04179 | CTD; DisGeNET; GeneCards; LLM | 3.49E-22 |
| 18 | BDNF | P23560 | CTD; DisGeNET; GeneCards; LLM | 3.48E-22 |
| 19 | CASP3 | P42574 | CTD; DisGeNET; GeneCards; LLM | 3.05E-22 |
| 20 | HMOX1 | P09601 | CTD; DisGeNET; GeneCards; LLM | 2.79E-22 |
| 21 | DRD1 | P21728 | CTD; DisGeNET; GeneCards; LLM | 2.39E-22 |
| 22 | ADORA2A | P29274 | CTD; GeneCards; TTD; LLM | 2.00E-22 |
| 23 | GFAP | P14136 | CTD; DisGeNET; GeneCards; LLM | 1.83E-22 |
| 24 | ATP13A2 | Q9NQ11 | CTD; DisGeNET; GeneCards; LLM | 1.67E-22 |
| 25 | MAPK1 | P28482 | CTD; DisGeNET; GeneCards; LLM | 1.67E-22 |
| 26 | MAPK3 | Q16644 | CTD; DisGeNET; GeneCards; LLM | 1.64E-22 |
| 27 | NGF | P01138 | CTD; DisGeNET; GeneCards; LLM | 1.63E-22 |
| 28 | IL1B | P01584 | CTD; DisGeNET; GeneCards; LLM | 1.59E-22 |
| 29 | SLC18A2 | Q05940 | CTD; DisGeNET; GeneCards; LLM | 1.56E-22 |
| 30 | CASP9 | P55211 | CTD; DisGeNET; GeneCards; LLM | 1.50E-22 |

**Table S5.** Chemical constituents of *Ginkgo biloba* with relative abundance rankings and normalized content weights.

| Arearank^a^ | Pubchem ID^b^ | Normalized Abundance^c^ | Arearank | PubChem ID | Normalized Abundance |
| --- | --- | --- | --- | --- | --- |
| 1 | 368156 | 1 | **46** | 174862 | 0.021739 |
| 2 | 161306 | 0.5 | **47** | 5462193 | 0.021277 |
| 3 | 9909368 | 0.333333 | **48** | 5280863 | 0.020833 |
| 4 | 5281696 | 0.25 | **49** | 5318118 | 0.020408 |
| 5 | 65243 | 0.2 | **50** | 5480982 | 0.02 |
| 6 | 5481663 | 0.166667 | **51** | 7427 | 0.019608 |
| 7 | 5280442 | 0.142857 | **52** | 5280704 | 0.019231 |
| 8 | 6324617 | 0.125 | **53** | 6916254 | 0.018868 |
| 9 | 5280805 | 0.111111 | **54** | 5318645 | 0.018519 |
| 10 | 5280794 | 0.1 | **55** | 382073 | 0.018182 |
| 11 | 5271805 | 0.090909 | **56** | 16066 | 0.017857 |
| 12 | 155892 | 0.083333 | **57** | 5281811 | 0.017544 |
| 13 | 9867869 | 0.076923 | **58** | 5281699 | 0.017241 |
| 14 | 5318569 | 0.071429 | **59** | 5281702 | 0.016949 |
| 15 | 44575467 | 0.066667 | **60** | 73581 | 0.016667 |
| 16 | 1094 | 0.0625 | **61** | 5316891 | 0.016393 |
| 17 | 5281854 | 0.058824 | **62** | 12304738 | 0.016129 |
| 18 | 5281665 | 0.055556 | **63** | 5318767 | 0.015873 |
| 19 | 5281617 | 0.052632 | **64** | 10604651 | 0.015625 |
| 20 | 76581 | 0.05 | **65** | 21577860 | 0.015385 |
| 21 | 44575480 | 0.047619 | **66** | 5280445 | 0.015152 |
| 22 | 5280443 | 0.045455 | **67** | 11025164 | 0.014925 |
| 23 | 439246 | 0.043478 | **68** | 5281858 | 0.014706 |
| 24 | 5281654 | 0.041667 | **69** | 9840292 | 0.014493 |
| 25 | 11664897 | 0.04 | **70** | 370 | 0.014286 |
| 26 | 6199 | 0.038462 | **71** | 637542 | 0.014085 |
| 27 | 11154476 | 0.037037 | **72** | 5281377 | 0.013889 |
| 28 | 5281852 | 0.035714 | **73** | 122850 | 0.013699 |
| 29 | 5281614 | 0.034483 | **74** | 6473881 | 0.013514 |
| 30 | 12308753 | 0.033333 | **75** | 10191345 | 0.013333 |
| 31 | 5280343 | 0.032258 | **76** | 10073778 | 0.013158 |
| 32 | 5281697 | 0.03125 | **77** | 439533 | 0.012987 |
| 33 | 72 | 0.030303 | **78** | 160521 | 0.012821 |
| 34 | 5316673 | 0.029412 | **79** | 11289099 | 0.012658 |
| 35 | 5315459 | 0.028571 | **80** | 11168362 | 0.0125 |
| 36 | 247 | 0.027778 | **81** | 5316262 | 0.012346 |
| 37 | 5281600 | 0.027027 | **82** | 10167806 | 0.012195 |
| 38 | 10169367 | 0.026316 | **83** | 11259090 | 0.012048 |
| 39 | 135 | 0.025641 | **84** | 5280656 | 0.011905 |
| 40 | 14448072 | 0.025 | **85** | 124219 | 0.011765 |
| 41 | 10228 | 0.02439 | **86** | 14033813 | 0.011628 |
| 42 | 5378597 | 0.02381 | **87** | 10975022 | 0.011494 |
| 43 | 5281612 | 0.023256 | **88** | 72307 | 0.011364 |
| 44 | 5273570 | 0.022727 | **89** | 5282160 | 0.011236 |
| 45 | 440752 | 0.022222 |  |  |  |

This table lists the bioactive chemical constituents identified in *Ginkgo biloba* from Meta-TCM. Only compounds indexed in the PubChem database with known or predicted protein interactions (BATMAN-TCM2.0) are included.

a: The ranking of the compound based on its relative abundance (peak area) derived from LC-MS analysis.

b: The unique compound identifier in the PubChem database.

c: The relative abundance weight of the compound, normalized to the range of [0, 1] relative to the most abundant constituent.
